# Supplementary material for: Networks of HIV-1 Envelope Glycans Maintain Antibody Epitopes in the Face of Glycan Additions and Deletions
Source: Structure. 2020 Aug 4;28(8):897–909.e6. doi: 10.1016/j.str.2020.04.022 (PMC7416112; doi:10.1016/j.str.2020.04.022)
Supplement: Document S1. Figures S1–S5 [file mmc1.pdf]

Structure, Volume *28*

Supplemental Information

Networks of HIV-1 Envelope Glycans Maintain Antibody Epitopes in the Face of Glycan Additions and Deletions

Gemma E. Seabright, Christopher A. Cottrell, Marit J. van Gils, Alessio D'addabbo, David J. Harvey, Anna-Janina Behrens, Joel D. Allen, Yasunori Watanabe, Nicole Scaringi, Thomas M. Polveroni, Allison Maker, Snezana Vasiljevic, Natalia de Val, Rogier W. Sanders, Andrew B. Ward, and Max Crispin

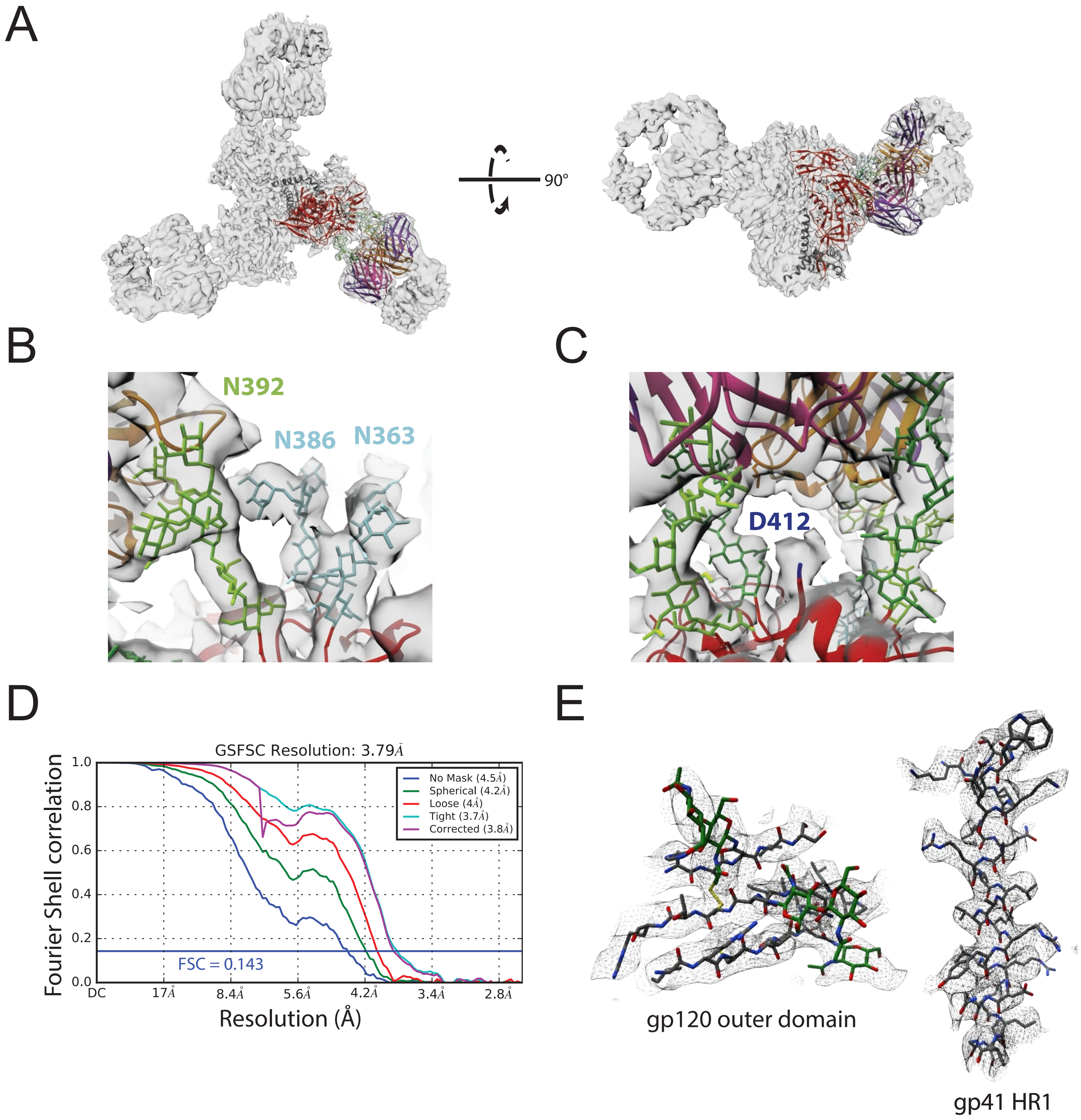

Figure S1. Cryo-EM of BG505 SOSIP.664 and 2G12 Fab2. Related to Figure 1. A) 3.8 Å cryo-EM 3D reconstruction and refined atomic model of BG505 SOSIP.664 with 2G12 Fab2. B) The close proximity of the N363 and N386 glycans to the N392 glycan could provide support for 2G12 binding via glycan/ glycan interactions. C) No coordinated density was seen for the N411 amino acid or the associated N- linked glycan. D) Fourier shell correlation (FSC) curves calculated in cryoSPARC during final non- uniform refinement. E) EM densities with atoms shown for a beta sheet in the gp120 outer domain and an alpha helix for the gp41 HR1 region. Related to Figure 1.

A

	N88	N130	N133	N136	N137	N141	N156	N160	N185e	N187	N188	N190	N190c	N197	N230	N234	N241	N262	N276	N289	N295	N301	N332	N339	N355
A	99	47	14	11	22	17	98	94	7	33	6	4	12	99	3	80	96	100	97	22	54	94	66	63	5
B	99	73	10	23	25	17	96	89	6	22	45	2	8	98	26	72	97	100	97	67	79	95	85	70	4
C	98	54	28	9	25	12	95	92	6	41	8	2	8	98	78	84	97	98	92	79	20	95	77	71	4
D	98	71	3	22	32	9	91	94	10	34	38	3	17	98	17	84	95	99	91	87	76	73	80	75	1
F	98	52	13	10	25	16	95	90	6	29	8	2	11	98	10	83	95	100	98	94	95	95	75	79	6
G	98	62	27	11	28	11	99	94	4	27	23	2	21	99	4	80	97	99	97	79	48	98	70	16	5
AE	98	66	14	3	14	16	98	93	8	39	6	2	6	98	0	86	99	100	95	96	85	79	2	47	4
Total	99	64	16	15	24	15	96	91	6	31	26	2	9	98	37	78	97	99	95	72	60	93	72	67	4

	N356	N362	N363	N386	N392	N396	N398	N406	N407	N411	N442	N448	N461	N462	N611	N616	N618	N625	N637
A	82	22	42	85	82	22	39	5	5	51	6	93	27	30	98	95	2	95	96
B	82	59	5	83	83	32	38	7	6	42	4	92	22	30	99	89	7	97	97
C	74	19	3	88	72	24	27	4	4	44	73	74	18	29	98	92	4	97	97
D	17	10	3	91	79	22	20	7	6	38	2	98	27	39	99	95	2	94	93
F	84	62	0	84	73	2	8	0	0	65	43	71	16	37	100	95	0	100	95
G	32	52	0	92	68	8	22	0	0	49	59	87	28	32	100	95	2	97	98
AE	75	1	0	93	77	15	43	1	1	64	7	95	31	25	98	98	0	97	98
Total	76	37	6	86	78	26	34	5	5	46	27	87	22	29	99	92	5	97	97

0 – 100 %

B

	Mean
A	28.9
B	30.5
C	29.1
D	29.2
F	29.7
G	30.0
AE	28.6

C

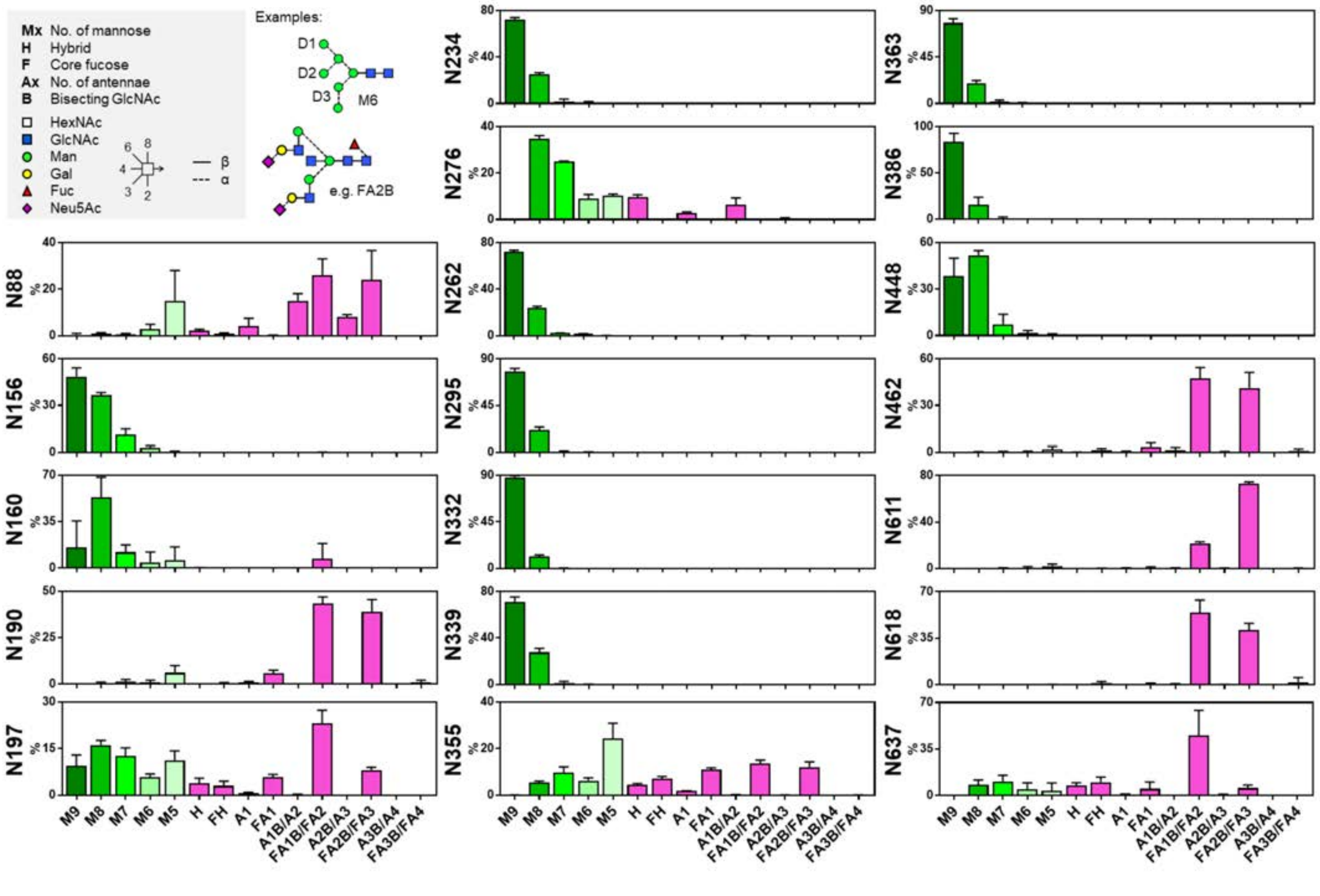

Figure S2. Conservation of PNGS across clades and reproducibility of LC-MS glycopeptide analysis. Related to Figures 1, 3, 4 and 5. A) Percentage conservation of individual PNGS. B) Mean number of total PNGS. The analysis was performed using sequence data obtained from the 2017 filtered web alignment from the Los Alamos HIV sequence database (https://www.hiv.lanl.gov). Clade A, n = 338; clade B, n = 2351; clade C, n = 1524; clade D, n = 127; clade F, n = 63; clade G, n = 114, clade AE, n = 535. Only the second position Asn was included for NN[S/T][S/T] motifs. Sites are only shown if present in one of the following representative strains: BG505 (clade A), AMC011 (clade B), CZA97 (clade C). The low conservation of the N332 site in clade AE is compensated by the high use of the N334 site (92%, AE; 21%, total). C) Relative quantification of glycan sites from three biological replicates of BG505 SOSIP.664. Error bars represent the standard error of the mean. The inset key depicts the glycan nomenclature and classification system used throughout this manuscript. HexNAc = N-acetylhexosamine, GlcNAc = N-acetylglucosamine, Man = mannose, Gal = galactose, Fuc = fucose, Neu5Ac = sialic acid. Glycan nomenclature according to the Consortium for Functional Glycomics, with linkages as per the Oxford system. Related to Figure 2.

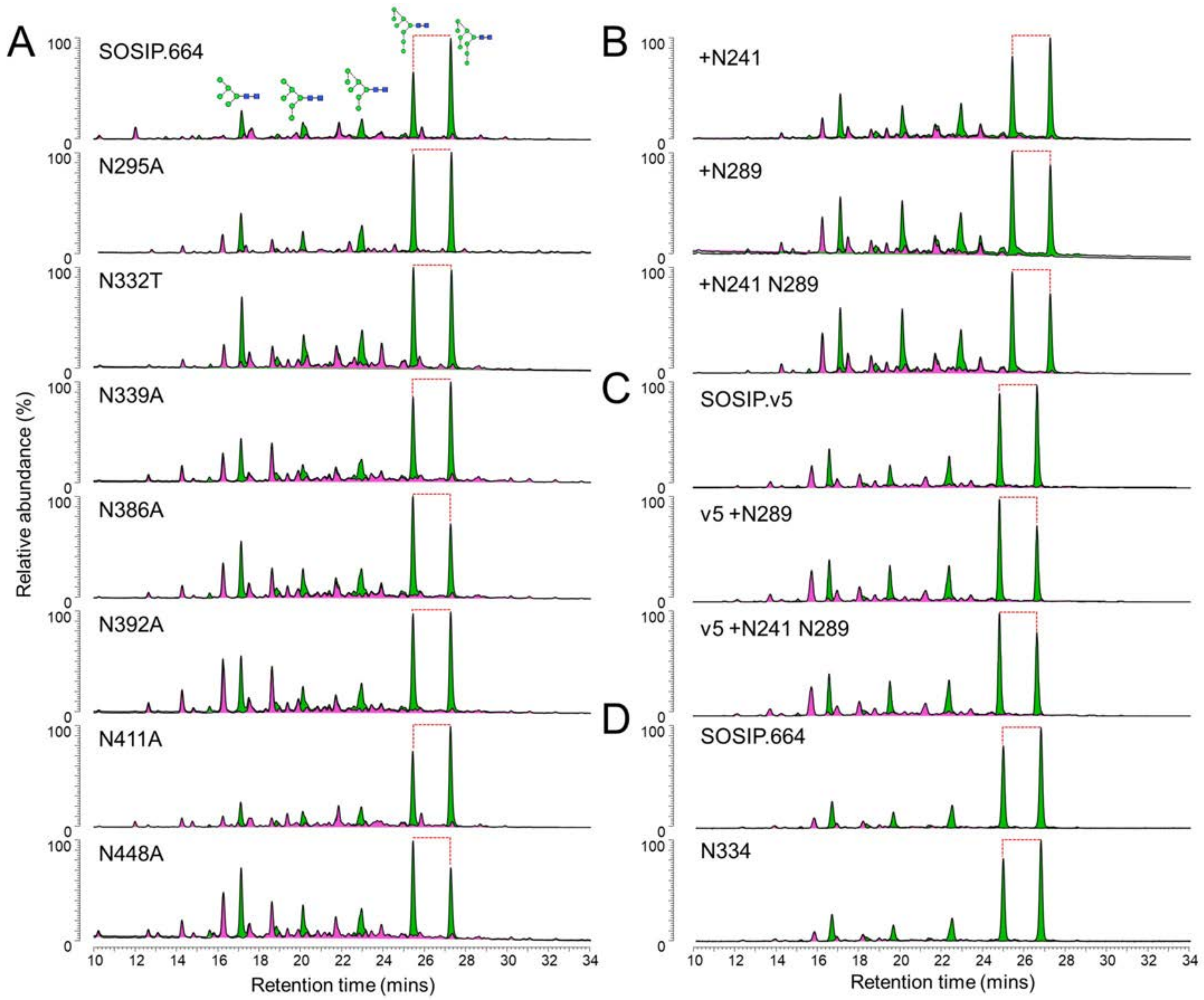

Figure S3. Glycosylation profiles of glycan mutants. Related to Figure 2. Hydrophilic interaction liquid chromatography-ultra performance liquid chromatography of PNGase F-released N-linked glycans from A) BG505 SOSIP.664 knockouts, B) BG505 SOSIP.664 knock-ins, C) BG505 SOSIP.v5 glycan knock-ins, and D) N334 shift mutation. Oligomannose-type glycans (judged by sensitivity to Endo H digestion) are highlighted in green. Complex-type glycans are shown in pink. Related to Figure 2.

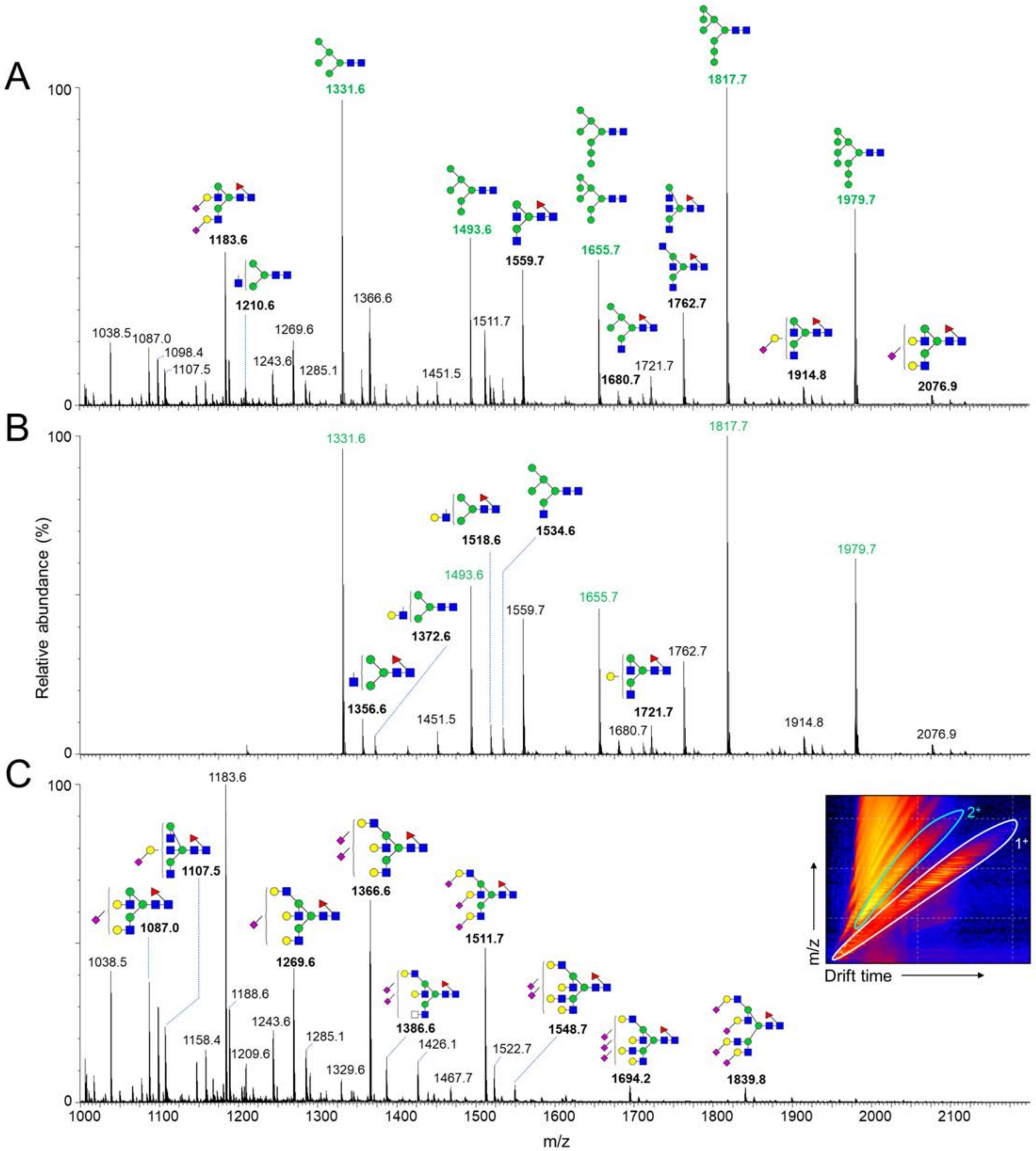

Figure S4. Generation of a glycan library. Related to Figures 3, 4 and 5. A) A glycan library was generated by negative ion electrospray ion mobility-mass spectrometry of an aliquot of unlabelled N-glycans from BG505 SOSIP.664 trimers. B) Mobility-extracted singly charged negative ions. C) Mobility-extracted doubly charged negative ions. The corresponding ions are encircled in white and blue, respectively, in the inset ion mobility drift plot. Related to Figure 3.

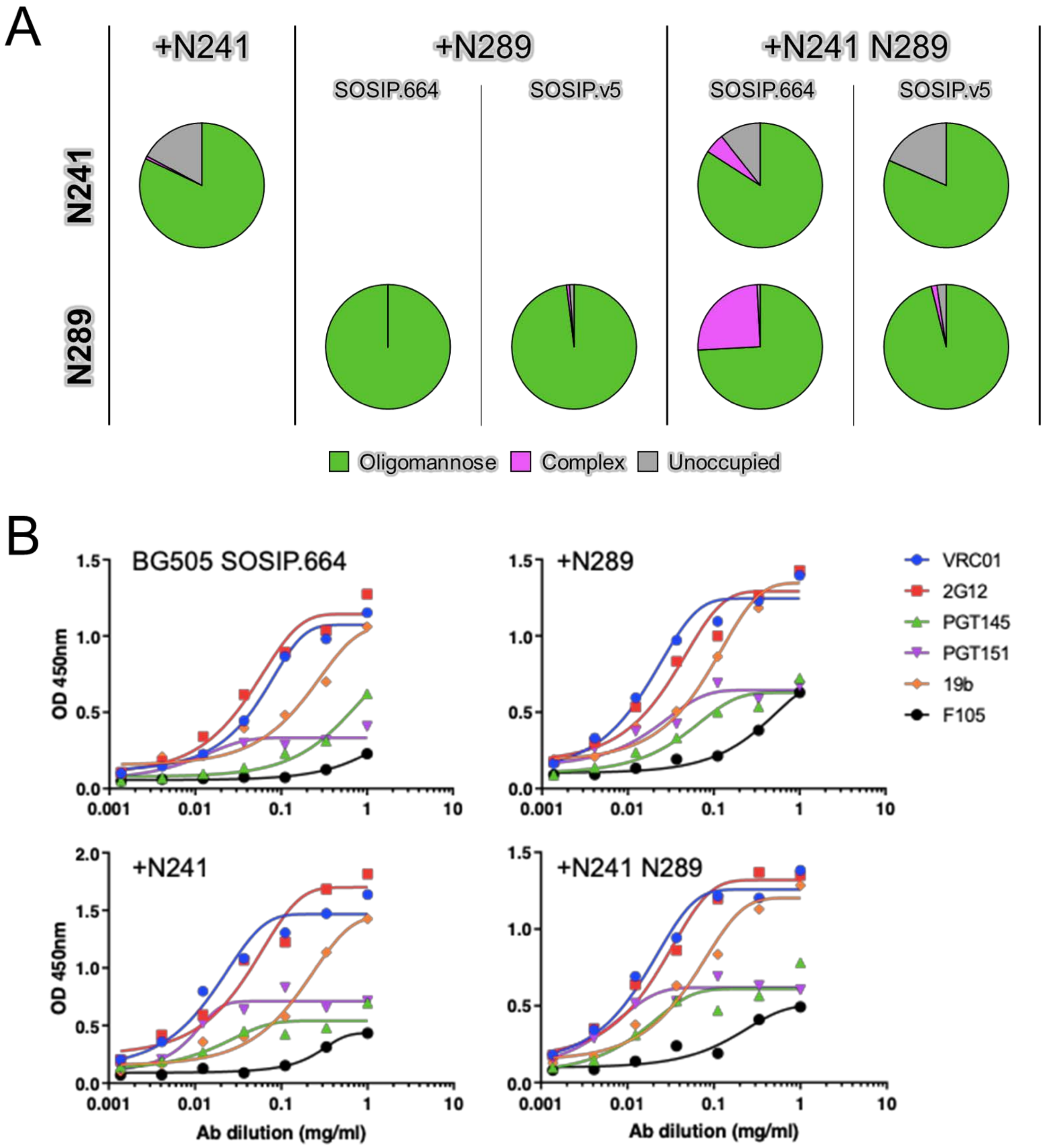

Figure S5. Classification of glycan-type at the N241 and N289 sites. Related to Figure 4. A) Relative quantification of the glycan-type occupying the N241 and N289 sites in the SOSIP.664 and SOSIP.v5 knock-in mutants. Glycopeptides were digested with Endo H to cleave oligomannose-type glycans, leaving behind a GlcNAc residue. The remaining complex-type glycans were then digested with PNGase F in the presence of ^{18}O-labeled water, to convert the Asn to an ^{18}O-labeled Asp, prior to LC-MS analysis, B) The BG505 SOSIP.664 glycan knock-ins are antigenically similar, as assessed by ELISA binding to a panel of neutralizing antibodies. Related to Figure 4.
