# Supplementary material for: Networks of HIV-1 Envelope Glycans Maintain Antibody Epitopes in the Face of Glycan Additions and Deletions
Source: Structure. 2020 Aug 4;28(8):897–909.e6. doi: 10.1016/j.str.2020.04.022 (PMC7416112; doi:10.1016/j.str.2020.04.022)
Supplement: Document S2. Article plus Supplemental Information [file mmc7.pdf]

Structure

Article

Networks of HIV-1 Envelope Glycans Maintain Antibody Epitopes in the Face of Glycan Additions and Deletions

Graphical Abstract

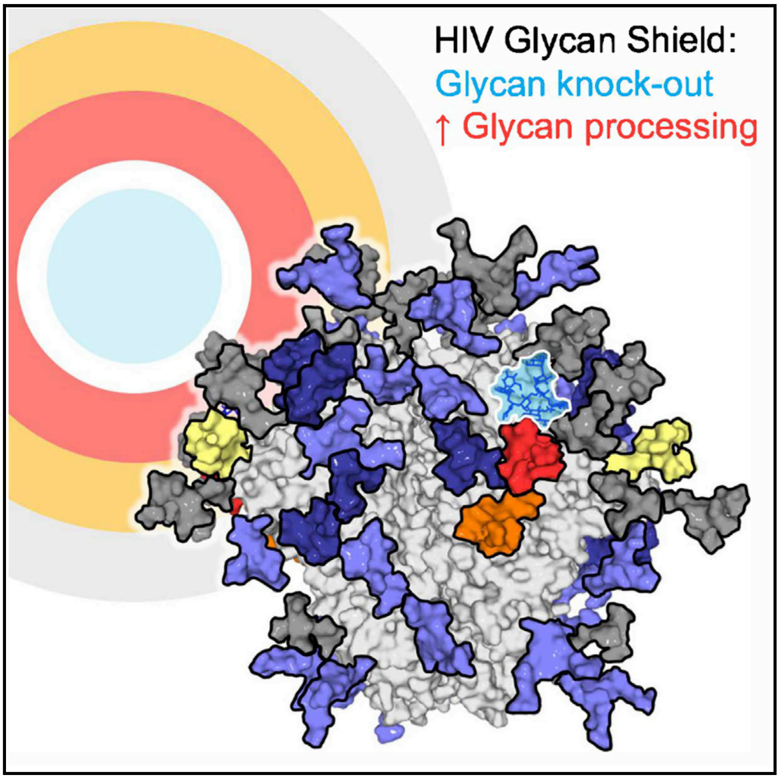

Authors

Gemma E. Seabright, Christopher A. Cottrell, Marit J. van Gils, ..., Rogier W. Sanders, Andrew B. Ward, Max Crispin

Correspondence

max.crispin@soton.ac.uk

In Brief

The human immunodeficiency virus is coated in a dense array of glycans that can be targeted by broadly neutralizing antibodies. Seabright et al. investigate how the network of glycans act to limit the biosynthetic processing of glycans and maintain glycan-based antibody epitopes.

Highlights

- HIV glycans form epitopes for broadly neutralizing antibodies
- High-resolution structure of 2G12 in complex with envelope glycoprotein mimetic
- Impact of glycan knockouts and knockins on glycan processing and epitope formation

Seabright et al., 2020, Structure *28*, 897–909
August 4, 2020 © 2020 The Author(s). Published by Elsevier Ltd.
https://doi.org/10.1016/j.str.2020.04.022

CellPress

Structure

CellPress
OPEN ACCESS

Article

Networks of HIV-1 Envelope Glycans Maintain Antibody Epitopes in the Face of Glycan Additions and Deletions

Gemma E. Seabright,[1,2,9] Christopher A. Cottrell,[3,9] Marit J. van Gils,[4] Alessio D'addabbo,[1] David J. Harvey,[1,5] Anna-Janina Behrens,[2] Joel D. Allen,[1] Yasunori Watanabe,[1,2] Nicole Scaringi,[3] Thomas M. Polveroni,[3] Allison Maker,[2] Snezana Vasiljevic,[2] Natalia de Val,[6,7] Rogier W. Sanders,[4,8] Andrew B. Ward,[3] and Max Crispin[1,2,10,*]

[1]School of Biological Sciences, University of Southampton, Southampton SO17 1BJ, UK
[2]Oxford Glycobiology Institute, Department of Biochemistry, University of Oxford, Oxford OX1 3QU, UK
[3]Department of Integrative Structural and Computational Biology, Scripps Research, La Jolla, CA 92037, USA
[4]Department of Medical Microbiology, Amsterdam UMC, AMC, University of Amsterdam, Amsterdam 1105 AZ, The Netherlands
[5]Target Discovery Institute, Nuffield Department of Medicine, University of Oxford, Old Road Campus, Oxford OX3 7FZ, UK
[6]Cancer Research Technology Program, Frederick National Laboratory for Cancer Research, Leidos Biomedical Research Inc., Frederick, MD 21701, USA
[7]Center for Molecular Microscopy, Center for Cancer Research, National Cancer Institute, National Institutes of Health, Bethesda, MD 20892, USA
[8]Department of Microbiology and Immunology, Weill Medical College of Cornell University, New York, NY 10021, USA
[9]These authors contributed equally
[10]Lead Contact
*Correspondence: max.crispin@soton.ac.uk
https://doi.org/10.1016/j.str.2020.04.022

SUMMARY

Numerous broadly neutralizing antibodies (bnAbs) have been identified that target the glycans of the HIV-1 envelope spike. Neutralization breadth is notable given that glycan processing can be substantially influenced by the presence or absence of neighboring glycans. Here, using a stabilized recombinant envelope trimer, we investigate the degree to which mutations in the glycan network surrounding an epitope impact the fine glycan processing of antibody targets. Using cryo-electron microscopy and site-specific glycan analysis, we reveal the importance of glycans in the formation of the 2G12 bnAb epitope and show that the epitope is only subtly impacted by variations in the glycan network. In contrast, we show that the PG9 and PG16 glycan-based epitopes at the trimer apex are dependent on the presence of the highly conserved surrounding glycans. Glycan networks underpin the conservation of bnAb epitopes and are an important parameter in immunogen design.

INTRODUCTION

The envelope spike (Env) of the human immunodeficiency virus type 1 (HIV-1) mediates infection of target host cells and is consequently a main target for vaccine design. However, Env displays extreme antigenic diversity, meaning only an immune response of exceptional breadth will be protective (Burton et al., 2012). In addition, a dense coat of host-derived, immunologically "self" N-linked glycans shield the underlying protein from host antibody responses (Wei et al., 2003). Despite these hurdles, approximately a third of infected individuals develop broadly neutralizing antibody (bnAb) responses against Env after several years of infection (Simek et al., 2009; van Gils et al., 2009).

Whereas the Env glycan shield typically limits antibody neutralization, many bnAbs have, paradoxically, evolved to recognize epitopes that are either entirely or partially composed of N-linked glycans (Blattner et al., 2014; Doores and Burton, 2010; Falkowska et al., 2014; Huang et al., 2014; McLellan et al., 2011; Pancera et al., 2013; Pejchal et al., 2011; Scharf et al., 2014; Walker et al., 2009, 2011). These bnAbs recognize the glycans at four distinct regions of Env: the gp120/gp41 protomer interface (e.g., PGT151), surrounding the CD4 binding site (e.g., HJ16), the V1/V2 loops at the trimer apex (e.g., PG9 and PG16), and the oligomannose-type glycans centered around the highly conserved N332 site on the outer domain of gp120 (e.g., PGT135 and 2G12) (Crispin et al., 2018). Given the number of bnAbs targeting the N332 glycan, it has previously been termed the "supersite of immune vulnerability" (Kong et al., 2013).

It is well established that the passive transfer of bnAbs protects non-human primates and humanized mice from viral challenge (Pegu et al., 2017; van Gils and Sanders, 2014). Thus, bnAbs are now being investigated for both therapeutic use

This is an open access article under the CC BY license (http://creativecommons.org/licenses/by/4.0/).

(Stephenson and Barouch, 2016), and to guide the design of Env-based immunogens intended to elicit similarly broad and neutralizing responses (Burton, 2017; Sanders and Moore, 2017). The latter approach typically involves producing recombinant mimics of the native, virion-associated Env trimer that present multiple bnAb epitopes, and/or immunogens specifically designed to target the germline-encoded bnAb precursors (gl-bnAbs) (Sanders and Moore, 2017; Stamatatos et al., 2017).

Currently, the most widely studied recombinant Env mimics are the BG505 SOSIP.664 trimers, based on the subtype A transmitted/founder virus sequence, BG505. Various modifications, including the introduction of a disulfide bond (SOS), an isoleucine to proline mutation (IP), and truncation at the 664 position (.664), increase both the stability and solubility of the trimers (Sanders et al., 2013). The resulting trimers display native-like structure and antigenicity (Sanders et al., 2013; Ward and Wilson, 2017) and are lead candidates in ongoing human immunogenicity studies (Dey et al., 2018; ClinicalTrials.gov Identifier: NCT03699241).

The BG505 transmitted/founder virus naturally lacks the conserved N332 glycan site, thus this glycan was also included (T332N) in the BG505 SOSIP.664 trimers to introduce the "supersite" epitope (Sanders et al., 2013). However, the BG505 sequence also lacks glycans at the 241 and 289 positions, despite their presence in 97% and 72% of HIV-1 isolates, respectively. The presence or absence of holes within the glycan shield has recently received a lot of attention because of the putative role of holes in initiating neutralizing antibody (nAb) responses or in redirecting the antibody response (Crooks et al., 2015, 2017; Gach et al., 2019; Klasse et al., 2016, 2018; Voss et al., 2017).

Immunization of rabbits with BG505 SOSIP.664 trimers elicits autologous nAb responses centered on glycan holes at positions 241 and 289 (Klasse et al., 2016; McCoy et al., 2016). Filling this hole (i.e., by introducing a glycan site) blocks antibody neutralization (McCoy et al., 2016). Similar results have been observed with nAbs targeting holes at the 130, 197, and 465 positions in immunogenicity studies with native-like trimers from different isolates (Crooks et al., 2015, 2017; Klasse et al., 2016, 2018; Voss et al., 2017). Such autologous nAb responses can be readily redirected *in vivo* by closing the glycan holes and opening new ones elsewhere on the trimer (Ringe et al., 2019). This phenomenon is echoed in natural infection, as the glycan shield "shifts" to escape arising nAbs (Dacheux et al., 2004; Moore et al., 2012; Wagh et al., 2018; Wei et al., 2003). The N332 glycan, for example, has been observed to shift from the N334 position and back again after the appearance of nAbs (Moore et al., 2012). While it is accepted that glycan holes offer an immunodominant distraction capable of eliciting autologous nAbs, the extent to which holes hinder the development of bnAbs remains largely unknown. There is evidence to suggest that more complete glycan shields in transmitted/founder viruses correlate with the development of greater neutralization breadth in infected individuals (Wagh et al., 2018). Future immunization strategies may, therefore, include immunogens with closed glycan holes, to redirect the nAb response away from the immunodominant protein surface toward more broadly neutralizing glycan-based epitopes (McCoy et al., 2016; Ringe et al., 2019).

The elicitation of a bnAb response requires the activation of bnAb precursor B cells. Effective immunogens must, therefore, be capable of engaging the B cell receptor (i.e., the gl-bnAb), before affinity maturation of the bnAb in the germinal centers. However, this process is hampered by the low affinity of gl-bnAbs to Env, often due to their inability to accommodate conserved N-linked glycans (Doores et al., 2013; Hoot et al., 2013; Ma et al., 2011; McGuire et al., 2014; Xiao et al., 2009). Thus an alternative, albeit closely linked, approach to eliciting bnAbs, is to prime with glycan-depleted immunogens capable of engaging gl-bnAbs, and subsequently boost with their "filled-in" derivatives to drive the development of neutralization breadth (Jardine et al., 2013; McGuire et al., 2013; Medina-Ramirez et al., 2017; Stamatatos et al., 2017; Steichen et al., 2016).

Glycan density, however, impacts glycosylation processing, which can in turn influence epitope presentation. The unusually high density of N-linked glycans on gp120 limits the extent to which individual sites can be processed by the host's α-mannosidases (Behrens and Crispin, 2017). Thus, gp120 displays a significant population of under-processed oligomannose-type glycans, termed the intrinsic mannose patch (IMP) (Bonomelli et al., 2011; Doores et al., 2010a; Go et al., 2013; Pritchard et al., 2015a). Analysis of recombinant, monomeric gp120 revealed that the removal of individual glycan sites from within the IMP often results in larger-than-expected decreases in the abundance of oligomannose-type glycans, as sites surrounding the deletion become more susceptible to glycan processing (Pritchard et al., 2015a). In Env trimers displaying native-like conformations, additional steric hindrances imposed by glycan and protein elements from neighboring protomers give rise to a further trimer-associated mannose patch (Behrens et al., 2017a; Cao et al., 2017; Pritchard et al., 2015c). Analysis of glycan-depleted, trimeric immunogens also revealed increased glycan processing at sites proximal to the glycan deletions (Behrens et al., 2018; Cao et al., 2017). Furthermore, correlations between glycan density and the abundance of under-processed oligomannose-type glycans have been reported (Coss et al., 2016; Stewart-Jones et al., 2016). Thus, while oligomannose-type glycans are a conserved feature of the Env glycan shield, and a key bnAb target, in some circumstances they can become susceptible to enzymatic processing.

Given the propensity for glycan density to influence the processing of glycans, we sought to determine the impact of individual glycan site additions and deletions on bnAb epitopes. Here, using glycopeptide analysis of BG505 SOSIP.664 trimers, we reveal that glycan site addition and deletion influences the fine processing of glycans both proximal to the mutated glycan site and elsewhere on the trimer. We further probe the tolerance of bnAbs to glycan mutations, and reveal the differing dependencies of mannose patch-targeting and apex-targeting bnAbs on the surrounding N-linked glycan sites. We also report a high-resolution structure of the 2G12 bnAb in complex with the BG505 SOSIP.664 trimer by cryo-electron microscopy (cryo-EM) and reveal details of the wider network of glycans that maintain the epitope. Furthermore, we show the N334 to N332 escape mutation minimally impacts glycosylation processing. The diverse impact of glycan holes on glycan-dependent bnAbs underscores the role of glycopeptide analysis in vaccine design and the development of new immunogens.

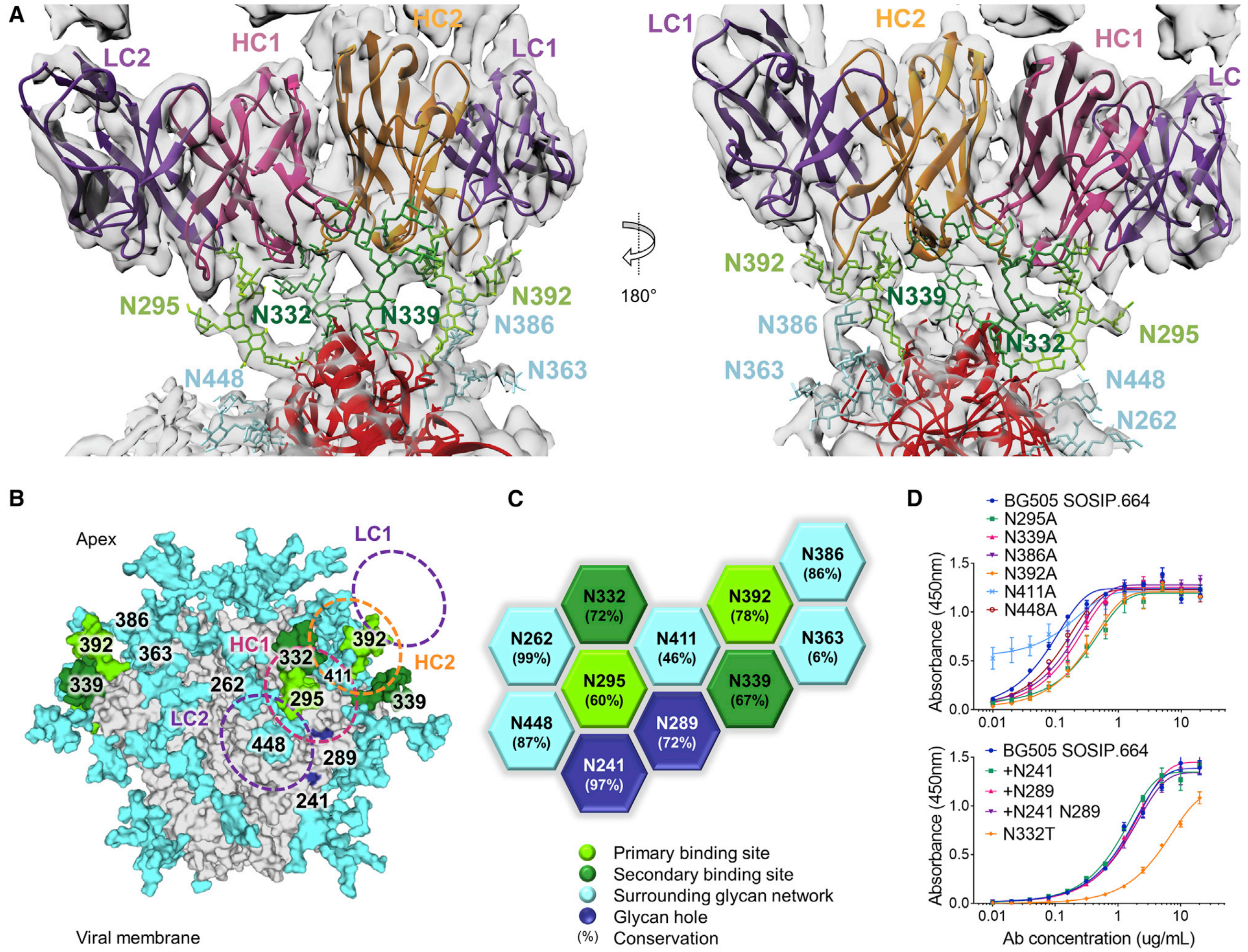

Figure 1. The 2G12 Epitope

(A) The cryo-EM structure of the 2G12 Fab_2 in complex with BG505 SOSIP.664 was resolved to 3.8 Å. Electron density is shown in gray. No density was observed for the glycan at N411 (Figure S1). Glycans bound by the 2G12 primary binding site are colored light green, glycans bound by the secondary binding site are colored dark green, the surrounding network of glycans are shown in cyan.

(B) Model of a fully glycosylated BG505 SOSIP.664 trimer based on PDB: 5ACO with glycans added according to Behrens et al., 2016 . The glycan holes at the 241 and 289 positions are highlighted in dark blue. The footprint of 2G12 is shown for orientation.

(C) The network of glycans surrounding the 2G12 epitope, and their conservation (Figure S2).

(D) 2G12 binding to glycan knockouts and knockins was assessed by ELISA, mean ± SEM.

RESULTS

Enhanced Mapping of the 2G12 Epitope by Cryo-EM

To elucidate the molecular details of the 2G12 epitope, we solved a 3.8 Å structure of 2G12 Fab_2 bound to BG505 SOSIP.664 by cryo-EM (Figures 1A and S1; Table S1). The two Fabs of the 2G12 bnAb are known to adopt a domain-swapped dimer conformation (Fab_2) via exchange of their VH domains (Calarese et al., 2003; Doores et al., 2010b). This unique architecture creates two primary binding sites at the VH/VL interfaces and two secondary binding sites on either side of the VH/VH interface. The 2G12 Fab_2 binds to a 1,879 $Å^2$ epitope composed entirely of N-linked glycans (Calarese et al., 2005; Sanders et al., 2002; Scanlan et al., 2002; Trkola et al., 1996). The primary binding sites make contact with the terminal α1,2-linked mannose residues of the D1-arms of the oligomannose-type glycans at positions N392 and N295 (Figures 1A–1C; see Figure S2 for glycan nomenclature). The secondary binding sites make contact with the D2- and D3-arms of the oligomannose-type glycans at positions N332 and N339 (Figures 1A–1C). Although not directly in contact with the 2G12 Fab_2, the glycans at N363 and N386 may also play a role in 2G12 binding by providing support to the N392 glycan via glycan/glycan interactions (Figure S1B) (Sanders et al., 2008). Previous studies have reported a potential contact between the 2G12 Fab_2 and glycans at positions N137 and N411 (Chuang et al., 2019; Murin et al., 2014); however, no coordinated density was observed for these glycans in our reconstruction, indicating that they do not play a direct structural role in 2G12 binding (Figure S1C).

To further gauge the contribution of individual glycans to the 2G12 epitope, we assessed antibody binding to glycan knockouts by ELISA. As expected, the N295A and N392A deletions

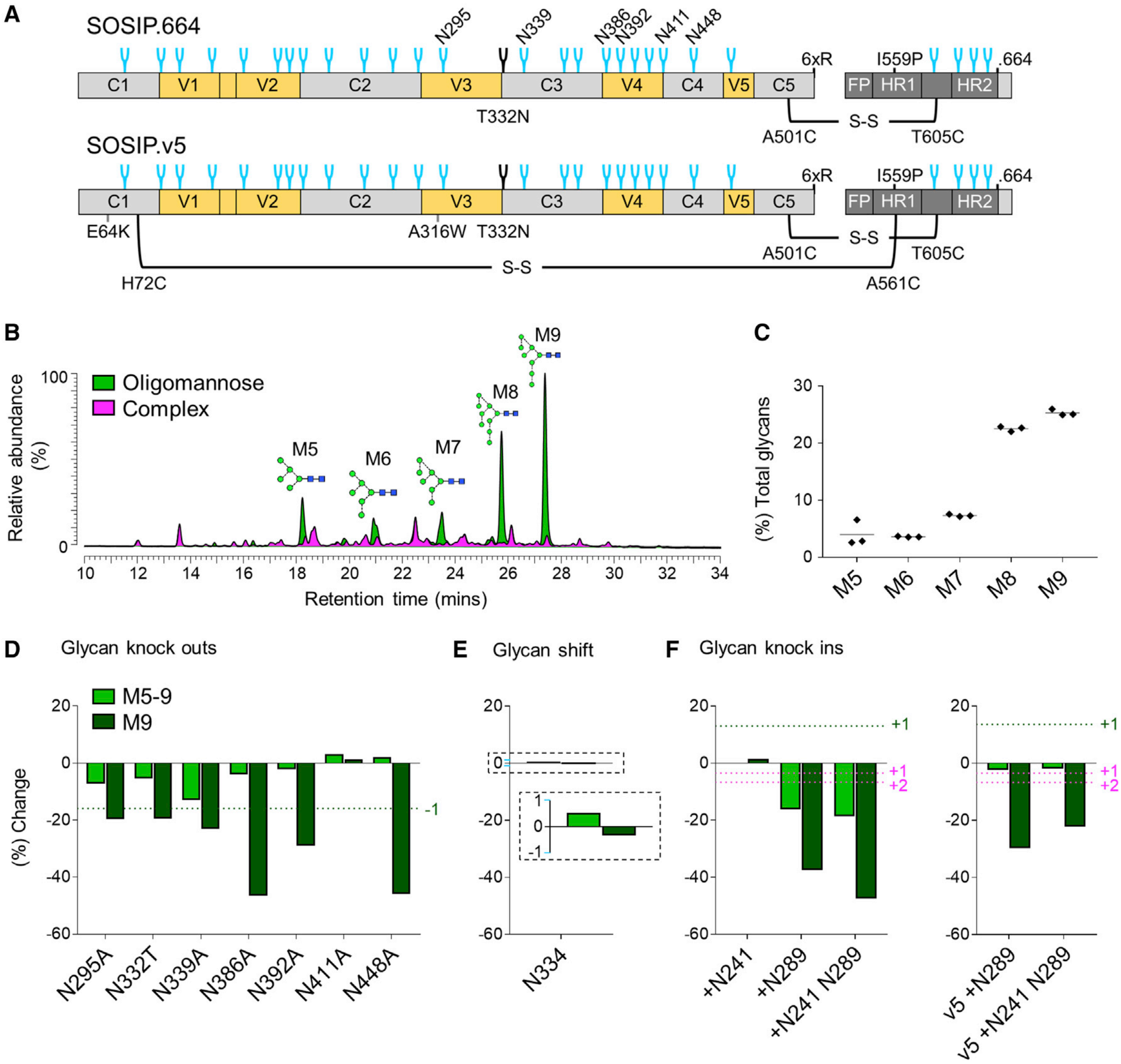

Figure 2. Impact of Glycan Site Additions and Deletions on Overall Glycosylation Profiles

(A) Linear schematic of the BG505 SOSIP.664 and SOSIP.v5 trimers, with stabilizing mutations annotated.

(B) Example HILIC-UPLC profile of fluorescently labeled N-linked glycans released from BG505 SOSIP.664. Oligomannose-type glycans (green) are quantified by integration of peaks before and after digestion with Endo H (pink).

(C) Quantification of individual oligomannose-type glycans from three biological replicates of BG505 SOSIP.664.

(D–F) Effect of glycan site deletion, shift, or addition on the abundance of Man9 (M9) and total oligomannose-type glycans (M5-9). Values represent the percentage change in abundance, relative to BG505 SOSIP.664: percentage change = ([% glycan mutant − % BG505 SOSIP.664]/% BG505 SOSIP.664) × 100. The dashed green lines represent the change in Man9 expected upon either the deletion (D) or addition (F) of a glycan site comprising solely Man9 structures. The dashed magenta line (F) represents the decrease in the abundance of Man9 expected upon the addition of one or two sites comprising solely complex-type glycans.

resulted in a substantial decrease in antibody binding (Figure 1D). In addition, deletion of the secondary binding site glycans, N332 and N339, reduced 2G12 binding. Of note, the N448A and N386A knockouts, while not directly contributing to the 2G12 epitope, diminished binding to a similar extent as the N339A secondary binding site deletion. In contrast, the N411A mutation increased 2G12 binding. The impact of glycan knockouts on 2G12 binding was also assessed by biolayer interferometry, and was in close agreement with the ELISA data (Table S2). Deletion of the N363 glycan had a very modest effect on 2G12 binding. In addition, we sought to determine the impact of filling the nearby 241/289 glycan hole on 2G12 binding. Neither the +N241 or +N289 glycan knockins, nor the +N241 N289 double knockin, had a substantial impact on 2G12 binding (Figure 1C).

Overall Resilience of the Mannose Patches to Glycan Addition and Deletion

To assess whether the observed differences in 2G12 binding could be attributed to differences in glycan processing, the glycosylation profiles of the glycan site mutants were compared with that of BG505 SOSIP.664 trimers (Figure 2). As the removal

of glycan sites can conceivably negatively impact trimer integrity, a quaternary structure-dependent antibody (PGT145), targeting a region distal to the site of mutation, was used for purification. The N-glycans from the target glycoproteins were enzymatically released, fluorescently labeled, and analyzed by hydrophilic interaction liquid chromatography-ultra performance liquid chromatography (HILIC-UPLC) (Figure S3). Quantification of oligomannose-type species was performed by the integration of chromatograms before and after digestion with endoglycosidase H (Endo H) (Figure 2B). In addition, the oligomannose-type glycans from three biological replicates of BG505 SOSIP.664 were quantified (Figure 2C), revealing a coefficient of variation of 2.2% for $Man_9GlcNAc_2$ and 1.5% for total oligomannose-type glycans.

As per BG505 SOSIP.664 trimers, the chromatograms of all the glycan mutants were dominated by oligomannose-type glycans, although the distribution of individual oligomannose-type glycans, particularly $Man_9GlcNAc_2$ and $Man_8GlcNAc_2$, varied slightly (hereafter referred to as Man9, Man8, etc.) (Figure S3). Previous site-specific glycan analyses of BG505 SOSIP.664 trimers have reported the N295, N332, N339, N386, N392, and N448 sites to be occupied by oligomannose-type glycans, predominantly Man9 (Behrens et al., 2016; Cao et al., 2017). Accordingly, deletion of each of these sites resulted in a decrease in the abundance of both total oligomannose-type glycans and Man9 structures (Figure 2D). The largest changes were observed for the N448A and N386A glycan knockouts, which resulted in a 46% and 47% decrease in the abundance of Man9, respectively. Many of the observed decreases were somewhat larger than the decrease predicted upon the loss of a glycan site comprising solely Man9 structure (16%; Figure 2B, dashed line). Thus, glycan site deletion on BG505 SOSIP.664 trimers can result in widespread increased glycosylation processing. This effect was not universally observed; for example, the N411A glycan site knockout had minimal impact on overall glycosylation processing despite its location at the center of the IMP (Figures 1 and 2D).

In addition, we investigated the impact of the N332 to N334 glycan "shift" escape mutation on glycosylation processing. In contrast to the deletion of the N332 site, the migration of the glycan to the N334 position had negligible impact on either the abundance of Man9 or total oligomannose-type glycans (Figure 2E).

Given that glycan site deletion generally resulted in increased glycosylation processing, and a glycan shift mutation did not impact glycosylation processing, we hypothesized that glycan site addition may restrict processing. Analysis of the +N241 glycan knockin, however, revealed only a minimal increase in the relative abundance of Man9 structures (2%; Figure 2F). In contrast, the +N289 and +N241 N289 knockins resulted in a decrease in both the abundance of oligomannose-type glycans and Man9 structures. This result may be expected if the knocked in sites were composed of predominantly complex-type glycans. However, the observed decrease in the abundance of Man9 (38% and 48%, respectively) far exceeds the predicted decreases upon the addition of one or two sites containing only complex-type glycans (3% and 7%, respectively; Figure 2F, dashed line). Thus, both glycan site deletions and additions, including the N289 site, appear to be increasing the glycosylation processing of the trimer.

We hypothesized that the BG505 SOSIP.664 trimer may be unaccommodating of the N289 glycan, and that its addition may be inducing conformational changes, which in turn influence glycosylation processing. We therefore repeated the analysis of the +N289 and +N241 N289 knockins on hyperstabilized BG505 SOSIP.v5 trimers (Figure 2A), which incorporate further stabilizing mutations, including an additional inter-subunit disulfide bond, and display reduced conformational flexibility (Torrents de la Pena et al., 2017). While the decrease in the abundance of Man9 on the SOSIP.v5 background was not as severe as on the SOSIP.664 trimers, it still exceeded the decrease predicted if the knocked in sites were composed of only complex-type glycans (Figure 2F, dashed line).

Glycan Deletion Increases Mannose Trimming throughout the Trimer

To elucidate the impact of glycan site mutations at the site-specific level, we performed in-line liquid chromatography-mass spectrometry (LC-MS) analysis of glycopeptides from the above glycan knockout and knockin constructs. To aid the assignment of glycopeptides, a glycan library was generated by ion mobility-mass spectrometry analysis of an aliquot of unlabeled N-glycans from the BG505 SOSIP.664 protein (Figure S4; Table S3). The use of one glycan library for the subsequent analysis of all the glycan site mutants is justified given the likeness of their HILIC-UPLC glycan profiles (Figure S3). The LC-MS methodology has been validated previously (Behrens et al., 2016). In addition, we performed glycopeptide analysis on three biological replicates of BG505 SOSIP.664 to confirm that observed differences were not due to experimental variation (Figure S2).

The deletion of glycan sites from the network of glycans surrounding the 2G12 epitope generally resulted in increased glycosylation processing at the immediately adjacent sites. This was most significant for the N332T, N411A, and N448A glycan deletions surrounding the N295 glycan site. The loss of each of these glycans resulted in a 61, 80, and 78 percentage point (pp) (the arithmetic difference between two percentages) decrease in Man9 at the N295 site, respectively, generally accompanied by a compensatory increase in Man5-8 structures (Figure 3). The effect was reciprocal, although less pronounced, with the N295A glycan knockout increasing processing at the N332 and N448 sites (18 and 37 pp decrease in Man9, respectively; Figure 3, N295A). Similarly, the N386A mutation resulted in a 42 pp decrease in Man9 at the adjacent N363 site, and the N392A knockout increased processing at the surrounding N339 and N386 sites by 30 and 35 pp, respectively (Figure 3, N386A).

Increased glycosylation processing, however, was not entirely limited to sites adjacent to the glycan knockout. In many instances, glycan site deletion resulted in increased processing emanating from the point of knockout. For example, the N332T knockout also increased processing at the N448 and N339 sites (40 and 13 pp decrease, respectively; Figure 3, N332T). As before, the effect appeared reciprocal, as the N448A knockout increased processing at the N332 site (21 pp decrease), located beyond the N295 glycan (Figure 3; N448A). In addition, the N234 glycan, which forms a small cluster with the N276 glycan distal glycan to the IMP, demonstrated increased processing upon the deletion of the N386, N392, or N448 sites (Figure 3).

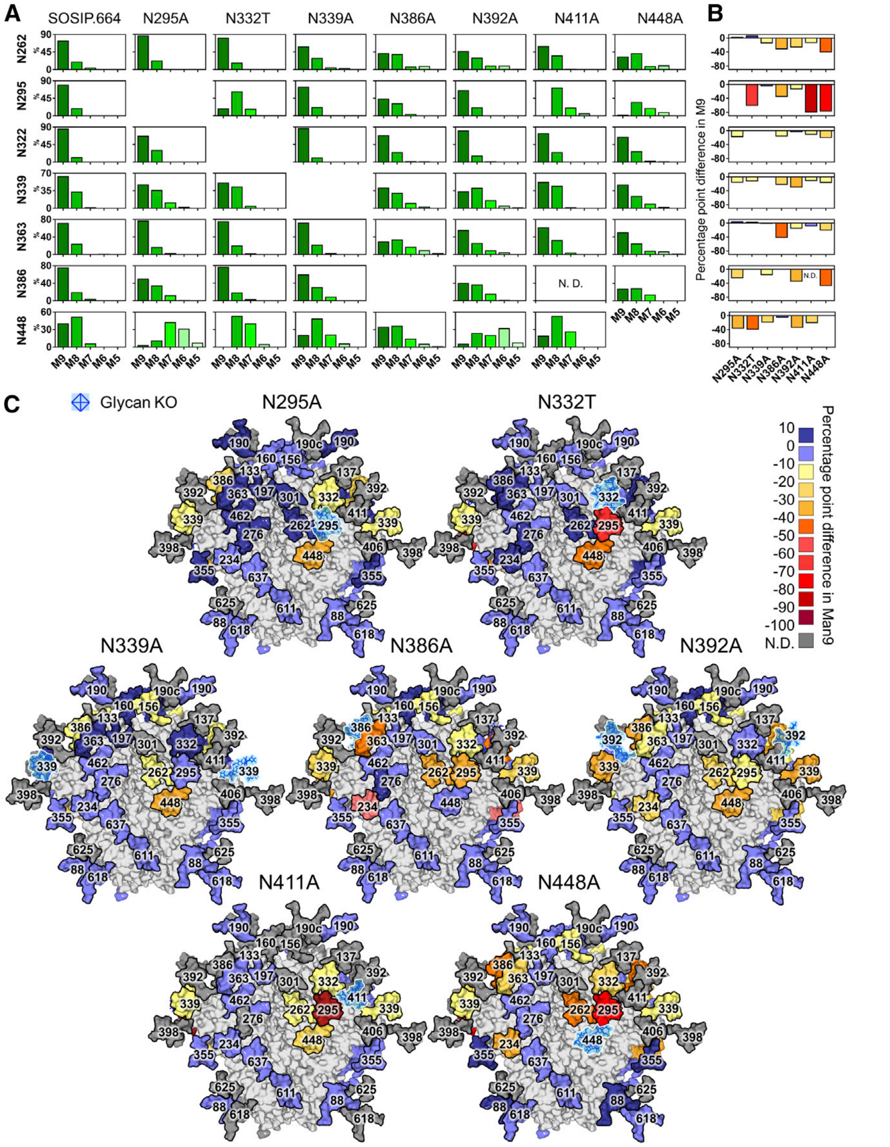

Figure 3. Glycan Deletion Increases Mannose Trimming throughout the Trimer

(A) Relative quantification of IMP sites from BG505 SOSIP.664 and the N295A, N332T, N339A, N386A, N392A, N411A, and N448A glycan knockouts. M9 = Man9 (dark green) to M5 = Man5 (pale green).

(B) The percentage point difference in the abundance of Man9 at IMP sites in the glycan knockouts, compared with BG505 SOSIP.664. Decreases in the abundance of Man9 are colored as per the key in (C).

(C) Heatmap demonstrating the percentage point difference in the abundance of Man9 at each site in the glycan knockouts, compared with BG505 SOSIP.664. Differences are calculated as follows: (% Man9 in knockout − % Man9 in BG505 SOSIP.664). KO, knockout; N.D., not determined.

Taken together, the results reveal the varying impact of glycan site knockouts on the processing of glycans across the glycan network, which may relate to underlying structural interactions between the glycans (Gristick et al., 2016; Lemmin et al., 2017; Stewart-Jones et al., 2016).

Differential Effects of Glycan Additions on Processing

In line with high glycan density limiting mannosidase trimming, glycopeptide analysis of the glycan knockin constructs revealed that the +N241 glycan site addition restricted processing at the neighboring N448 site, resulting in a 21 pp increase in the abundance of Man9 (Figure 4).

In contrast, the +N289 site knockin resulted in increased glycosylation processing, both at sites neighboring the introduced glycan and throughout the trimer. The N262, N295, and N332 sites all displayed a decrease in the abundance of Man9 (37, 20, and 28 pp decrease, respectively; Figure 4). The N363 and N234 sites were also affected to a similar extent, despite their greater distance from the +N289 knockin. The +N241 N289 double glycan knockin displayed both restricted glycosylation processing at the N448 site (12 pp increase in Man9) and increased processing at the N262, N295, N332, and N363 sites (Figure 4).

The increased glycan processing associated with the +N289 glycan knockin is surprising, given that high glycan density is generally associated with restricted glycosylation processing. We had hypothesized that the BG505 SOSIP.664 protein might be unable to accommodate a glycan at this site, and thus the addition of a glycan may be causing wider conformational changes to the protein. To address this, we assessed the binding of the knockin mutants to a panel of antibodies targeting distinct epitopes, and found the knockins to be antigenically similar to the BG505 SOSIP.664 protein (Figure S5B). In addition, we investigated the impact of the glycan knockins on the hyperstabilized SOSIP.v5 background (Torrents de la Pena et al., 2017) (Figure 2A). In line with the HILIC-UPLC analysis, glycopeptide analysis confirmed that the addition of the N289 glycan to the BG505 SOSIP.v5 background resulted in increased glycan processing, although to a slightly less extent than that observed on the SOSIP.664 background (Figure 4D). The SOSIP.v5 +N241 N289 double glycan knockin also exhibited changes in processing similar to that of the SOSIP.664 background, but not as pronounced.

We had considered that the decrease in the total abundance of oligomannose-type glycans observed by HILIC-UPLC analysis may be, at least partially, explained by the addition of a site(s) comprising predominantly complex-type glycans. However, the precise compositions of the N241 and N289 glycan additions could not be readily determined as they co-occupy peptides with the N234 and N295 sites, respectively. To classify the glycan type occupying these sites, we subjected the glycopeptides to sequential digests with Endo H (to cleave oligomannose-type

CellPress
OPEN ACCESS

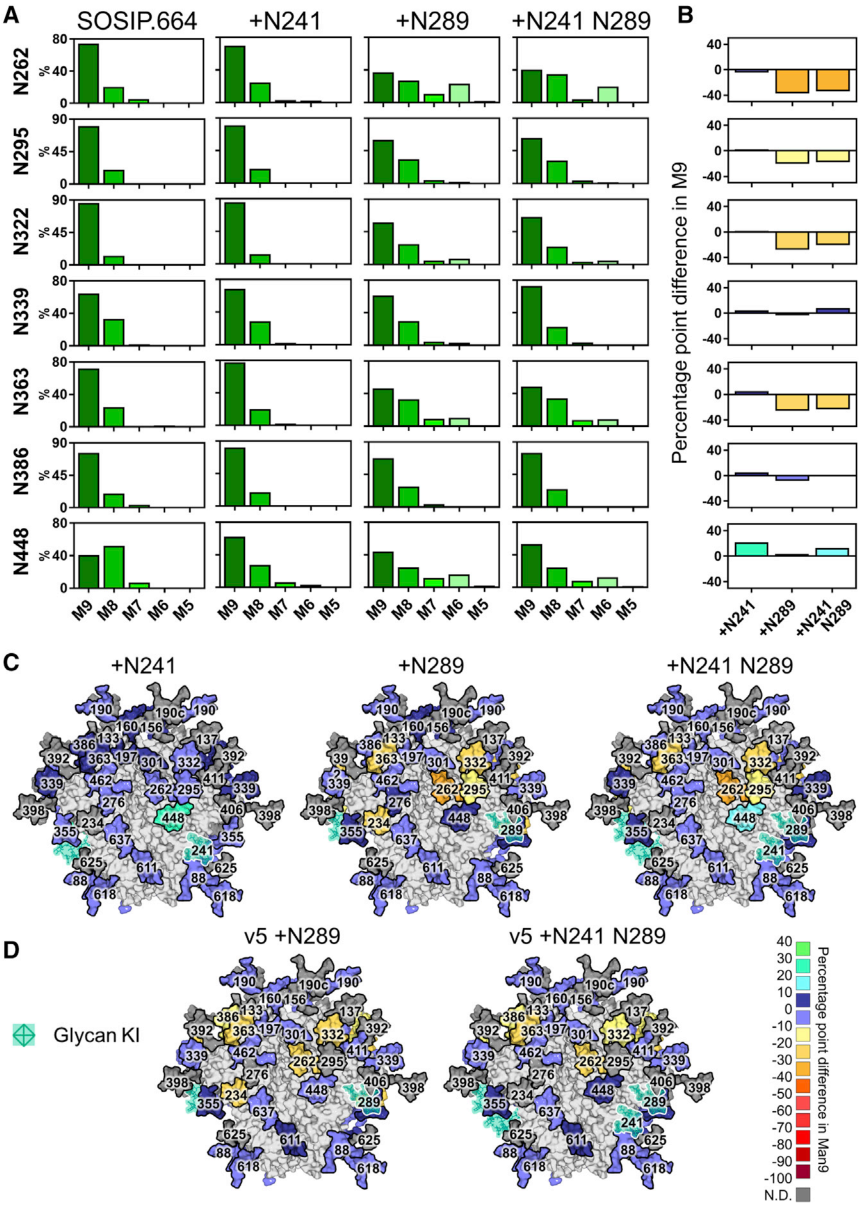

Figure 4. Differential Effect of Glycan Additions on Glycosylation Processing

(A) Relative quantification of IMP sites from BG505 SOSIP.664 and the +N241, +N289, and +N241 N289 glycan knockins. M9 = Man9 (dark green) to M5 = Man5 (pale green).

(B) The percentage point difference in the abundance of Man9 in the glycan knockins, compared with BG505 SOSIP.664. Graphs are colored according to the key in (D).

(C) Heatmap demonstrating the percentage point difference in the abundance of Man9 at each site in the glycan knockins, compared with BG505 SOSIP.664: (% Man9 in knockin − % Man9 in BG505 SOSIP.664).

(D) Percentage point difference in the abundance of Man9 at each site in the SOSIP.v5 glycan knockins compared with BG505 SOSIP.v5. KI, knockin.

glycans) and peptide-N-glycosidase F to cleave remaining complex-type glycans) (Cao et al., 2017). Both the N241 and N289 sites were found to be almost exclusively occupied by oligomannose-type glycans, irrespective of SOSIP.664 or SOSIP.v5 background (Figure S5), confirming that the observed decreases in the abundance of oligomannose-type glycans, particularly Man9 structures, are solely due to increased processing at other glycan sites on the trimer.

A Network of Glycans Preserves the PG9 and PG16 Epitopes

A large proportion of glycan-targeting bnAbs recognize the glycans of the V1/V2 loops, located at the trimer apex (Walker et al., 2009). This class of bnAbs is typified by the PG9 and PG16 antibodies, which contain very long heavy-chain complementarity-determining region 3, allowing for penetration of the N160 glycan triad at the trimer apex (Figure 5D) (McLellan et al., 2011; Pancera et al., 2013). The N160 glycan sits in the center of a network of glycans spanning all three protomers, including the highly conserved N156 and N197 sites (96% and 98%, respectively; Figure 5B).

In contrast to 2G12, which was largely tolerant of glycan site deletions, the binding of PG16 and, to a lesser extent, PG9 was significantly reduced upon the loss of the N156 glycan (Figure 5E). As before, this is somewhat expected as the N156 glycan directly contributes to the antibodies' epitopes (McLellan et al., 2011; Pancera et al., 2013). However, consistent with previous reports, the N197A glycan knockout also reduced antibody binding (Behrens et al., 2016). Given that the N197 glycan does not contribute to either antibody epitope, we hypothesized that the knockout may be disrupting glycosylation processing at the epitope and performed glycopeptide analysis to address this.

Removal of either the N156 or N197 glycan sites resulted in increased processing at the proximal N160 site (Figures 5A and 5C). This was particularly true of the N156A glycan knockout, which resulted in the complete processing of Man9 to smaller oligomannose-type structures and complex-type glycosylation, such that the dominant peak shifted from Man9 to afucosylated biantennary structures (Figure 5A). The N197A glycan knockout resulted in increased oligomannose trimming at the N160 site, resulting in Man8 predominating (Figure 5A). The N156A and N197A glycan knockouts also affected each other reciprocally, with each resulting in a slight loss of Man9 at the other site (13.3 and 16.4 pp, respectively; Figures 5A and 5B). We also note that the N363 site exhibited slightly increased oligomannose trimming upon the loss of both glycan sites, although the rest of the trimer appeared unaffected.

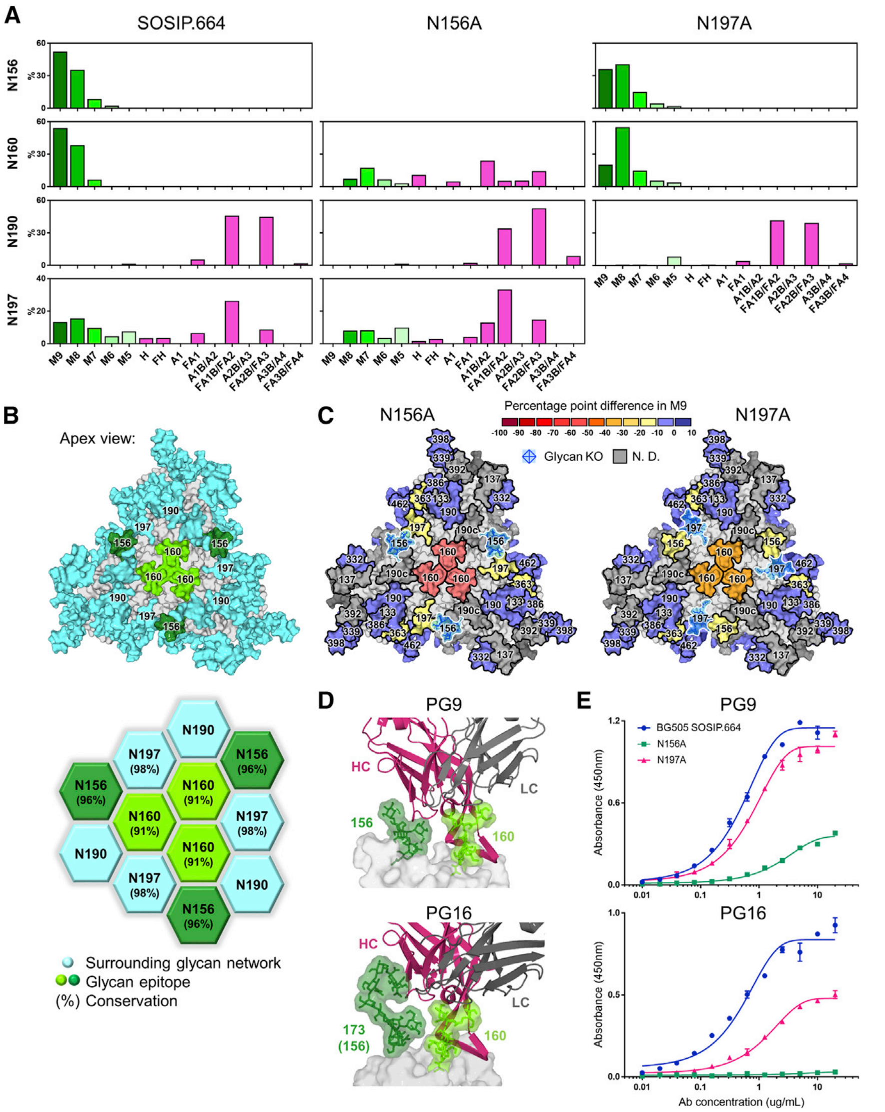

Figure 5. A Network of Glycans Preserves the PG9 and PG16 Epitopes

(A) Relative quantification of apex glycan sites on BG505 SOSIP.664 and N156A and N197A glycan knockouts. M9 = Man9 (dark green) to M5 = Man5 (pale green), complex-type glycans (magenta) are grouped according to their number of antenna (A1-4) and/or presence of a bisecting GlcNAc (B) and/or the presence of core fucose (F) (Figure S2).

(B) Model of a fully glycosylated trimer (top; as described in Figure 1) illustrating the network of glycans at the trimer apex (bottom).

(C) Heatmaps displaying the percentage point difference in Man9 on N156A and N197A glycan knockouts compared with BG505 SOSIP.664.

(D) Structure of the PG9 antibody in complex with the V1/V2 region of the CAP45 strain (PDB: 3U4E) and PG16 in complex with the V1/V2 region of ZM109 (PDB: 4DQO).

(E) ELISA data of PG9 and PG16 binding to BG505 SOSIP.664 and N156A and N197A glycan knockouts, mean ± SEM..

DISCUSSION

There are wide-ranging influences of glycan additions and deletions on HIV-1 immune evasion, both in the context of natural infection and in immunization regimens. At one level, the very high density of glycans on Env indicates a selective advantage in using glycans to evade elimination by the host immune system (Scanlan et al., 2007; Wyatt and Sodroski, 1998). However, the glycan shield does not simply evolve to a maximum number of glycans. Instead, the creation and filling of holes during the course of infection illustrates that active rearrangements are required for effective immune evasion (Dacheux et al., 2004; Moore et al., 2012; Wagh et al., 2018; Wei et al., 2003).

In the context of vaccination, the opening and closing of glycan holes in immunogens may prove a useful tool for driving the development of neutralization breadth (Jardine et al., 2013; McGuire et al., 2013; Medina-Ramirez et al., 2017; Ringe et al., 2019; Stamatatos et al., 2017; Steichen et al., 2016). The role of glycans in forming and blocking epitopes is of particular interest as bnAbs can evolve to recognize these structures (Moore et al., 2012). In some instances, the precise processing state of the glycan target is essential for bnAb recognition and neutralization, with glycan heterogeneity manifesting as <100% neutralization plateaus (Doores and Burton, 2010; Kong et al., 2013; McCoy et al., 2015; Pritchard et al., 2015b). The heterogeneity exhibited at a given glycan site can be influenced by the proximity of neighboring glycans (Behrens et al., 2018; Cao et al., 2017; Pritchard et al., 2015a). Using trimeric BG505 SOSIP.664 as a model system, we established the impact of individual glycan site mutations on the glycan networks at two key antigenic regions of the HIV-1 glycan shield, the IMP and the trimer apex.

The contribution of glycans to many bnAb epitopes has been defined by structural methods, such as X-ray crystallography and cryo-EM, complemented by glycopeptide analysis (Crispin et al., 2018; Ward and Wilson, 2017). In this study, we present the highest-resolution structure reported to date of 2G12 in complex with its Env target. We confirm that 2G12 directly contacts the oligomannose-type glycans at four sites and reveal the contributions of the surrounding glycans. It has previously been shown that mutations affecting glycan sites lying outside of bnAb epitopes can disrupt binding and/or neutralization (Behrens et al., 2016; Crispin et al., 2018; McCoy et al., 2016; Scanlan et al., 2002). In line with this, we reported that the deletion of glycan sites in the networks surrounding glycan-dependent antibody epitopes perturbed antibody binding, in some cases to the same extent as mutations directly impacting the epitope. Such observations may be partially explained by disruptions to the fine processing of the glycan epitope upon the mutation of

CellPress
OPEN ACCESS

proximal glycan sites, specifically increased trimming by mannosidases.

Despite causing significant disruption to the fine processing of the 2G12 epitope, the N411A knockout displayed increased 2G12 binding, consistent with previous reports (Scanlan et al., 2002). The N411 glycan was unresolved in the cryo-EM structure, although the asparagine residue occupied the middle of 2G12-glycan complex (Figure S1). A glycan at this site could potentially result in a clash or the entropy of the glycan could be reduced upon 2G12 binding (Figure 1). We also note the apparent discrepancy between the N411A mutation having minimal impact on the overall glycosylation profile while increasing processing at local sites. Such an effect may be explained by unresolved compensatory effects of other glycans.

The current study was restricted to the analysis of soluble BG505 SOSIP.664 immunogens, an important experimental model for viral glycosylation. We note that soluble SOSIP.664 trimers display somewhat increased oligomannose levels compared with virion-derived Env (Cao et al., 2018; Struwe et al., 2018). It could, therefore, be argued that mutations that increase the glycan processing of SOSIP.664 trimers are beneficial to generating immunogens that mimic the glycosylation of the native virus. However, oligomannose sites tend to be conserved between SOSIP.664 trimers and virion-derived Env (Cao et al., 2018; Struwe et al., 2018), thus the integrity of the mannose patch is associated with well-folded trimers. For example, within the BG505 SOSIP.664 experimental system, substantial changes in the abundance of oligomannose-type glycans can indicate deviations away from native-like conformations (Pritchard et al., 2015c).

In this context, we note that the mutation of N-linked glycan sites can induce glycoprotein misfolding or conformational changes (Kong et al., 2015; Pritchard et al., 2015a; Sanders et al., 2002; Wang et al., 2013). Here, the glycan mutants were purified using a quaternary structure-dependent antibody affinity step, PGT145 or PGT151 (Blattner et al., 2014; Walker et al., 2011), to minimize the contribution of misfolded proteins to the analysis. Accordingly, all the mutants displayed a glycosylation profile dominated by oligomannose-type glycans, a signature of native-like trimer configuration (Behrens and Crispin, 2017; Behrens et al., 2017a; Pritchard et al., 2015c). One potential caveat of using a glycan-dependent bnAbs for purification is the introduction of glycan bias mediated throughout the glycan network; however, previous comparisons of bnAb-purified BG505 SOSIP.664 proteins have found this effect to be negligible (Cao et al., 2017; Pritchard et al., 2015c).

The unexpected increase in glycosylation processing observed upon the knockin of the N289 glycan, in both the SOSIP.664 trimers and hyperstabilized SOSIP.v5 trimers, suggests that this mutation may be causing a degree of localized protein instability. We note that the +N289 knockin was generated by mutating the proline at the 291 position to serine. While it is impossible to completely unpick the impact of the proline mutation and the glycan addition, we note that the glycan knockins were antigenically similar to the parental protein. The results highlight the importance of characterizing the glycosylation of all candidate immunogens in depth (Behrens et al., 2017b).

Understanding the interdependence of glycans and their processing states is important in revealing how viral mutations can influence distant epitopes. Similarly, in immunogen design, the presence or absence of holes in the glycan shield could have wider antigenic and immunogenic consequences through glycan-glycan network effects. While the processing state of some glycans is key to the formation of bnAb epitopes (Kong et al., 2013; Pritchard et al., 2015b), the network may be dominated simply by the presence or absence of glycans independent of their processing state. Consistent with this view, we report that both the deletion of glycan sites surrounding the 2G12 epitope, and the addition of the N289 glycan, increased the mannosidase trimming of the epitope, but only glycan site knockouts had a measurable impact on 2G12 binding. This result suggests that the epitope is largely maintained by the surrounding network of glycans providing structural support to the four glycans directly involved in 2G12 binding, rather than through modulating the fine processing of the epitope. Indeed, previous studies have reported 2G12 binding to multiple oligomannose structures, including Man8 and the D1-isomer of Man7 (Dunlop et al., 2010).

The same network effects appear to apply to the epitopes of the apex binding antibodies, PG9 and PG16. Previous studies have reported the dependence of apex-targeting bnAbs on sialylated glycans at the N156 site (Andrabi et al., 2017; McLellan et al., 2011; Pancera et al., 2013). However, glycopeptide analyses presented both here and previously report this site to be predominantly occupied by oligomannose-type glycans (Behrens et al., 2016, 2017a; Cao et al., 2017, 2018). Thus, these bnAbs are able to tolerate glycan heterogeneity. The considerable loss of binding upon glycan site deletion is, therefore, symptomatic of the structural role the network of surrounding glycans plays in stabilizing this glycan epitope. At the apex in particular, the impact of the deletion of an individual glycan site may be amplified 3-fold.

It is not yet known the extent to which a successful vaccine candidate must display precise glycan epitopes. However, the results presented here shed light on the role of individual glycan sites in the fine processing of bnAb epitopes. We reveal the role of glycan networks in stabilizing the structure and fine processing of two key bnAb epitopes, the trimer apex and IMP. Future work should be directed to quantifying the contribution of individual glycoforms to antibody binding. A growing understanding of the factors shaping the glycosylation of Env will aid the continued development of HIV-1 immunogens.

STAR★METHODS

Detailed methods are provided in the online version of this paper and include the following:

- KEY RESOURCES TABLE
- RESOURCE AVAILABILITY
  - Lead Contact
  - Materials Availability
  - Data and Code Availability
- EXPERIMENTAL MODEL AND SUBJECT DETAILS
  - HEK 293F Cell Culture
- METHOD DETAILS
  - Glycan Site Mutagenesis

- EXPRESSION AND PURIFICATION OF PROTEINS
  - Cryo-Electron Microscopy and Model Building
  - Enzymatic Release of N-linked Glycans
  - Fluorescent Labeling of N-linked Glycans
  - HILIC-UPLC Analysis of the N-linked Glycans
  - Oligomannose-type Glycan Quantification
  - Ion Mobility-Mass Spectrometry of N-linked Glycans
  - Reduction, Alkylation and Digestion of Env Proteins
  - Liquid Chromatography-Mass Spectrometry Analysis of Glycopeptides
  - Site-Specific Glycan Classification
  - ELISAs
  - Biolayer Interferometry
- QUANTIFICATION AND STATISTICAL ANALYSIS

SUPPLEMENTAL INFORMATION

Supplemental Information can be found online at https://doi.org/10.1016/j.str.2020.04.022.

ACKNOWLEDGMENTS

This project has received funding from the European Union's Horizon 2020 Research and Innovation program under grant agreement no. 681137 (to R.W.S. and M.C.), the Bill and Melinda Gates Foundation through the Collaboration for AIDS Vaccine Discovery (OPP1084519 and OPP1115782 to M.C. and A.B.W. and OPP1132237 and INV-002022 to R.W.S.), the International AIDS Vaccine Initiative (IAVI) Neutralizing Antibody Consortium (NAC) (to M.C. and A.B.W.), the US NIH grant P01 AI110657 (to A.B.W. and R.W.S.), the National Institute of Allergy and Infectious Diseases Center for HIV/AIDS Vaccine Immunology and Immunogen Discovery (1UM1AI100663 to M.C. and A.B.W.), and the Consortium for HIV/AIDS Vaccine Development (UM1AI144462 to M.C. and A.B.W.). C.A.C. is supported by NIH F31 Ruth L. Kirschstein Predoctoral Award AI131873 and by the Achievement Rewards for College Scientists Foundation. M.J.v.G. is a recipient of a Mathilde Krim Fellowship from the American Foundation for AIDS Research (amfAR; grant 109514-61-RKVA). R.W.S. is a recipient of a Vici grant from the Netherlands Organisation for Scientific Research (NWO).

AUTHOR CONTRIBUTIONS

G.E.S., C.A.C., M.J.v.G., A.D., D.J.H., A.-J.B., N.D.V., N.S., T.M.P., A.M., and S.V. performed the experimental work. G.E.S., C.A.C., Y.W., and J.D.A analyzed the data. G.E.S. and M.C. wrote the paper. R.W.S., A.B.W., and M.C. designed the study. All authors read and approved the final manuscript.

DECLARATION OF INTERESTS

The International AIDS Vaccine Initiative (IAVI) has previously filed a patent relating to the BG505 SOSIP.664 trimer: US Prov. Appln. no. 61/772,739, entitled "HIV-1 Envelope Glycoprotein," with R.W.S. and A.B.W. among the co-inventors, but no patents have been filed on any work described here.

Received: May 13, 2019
Revised: February 27, 2020
Accepted: April 28, 2020
Published: May 19, 2020

REFERENCES

Agirre, J., Iglesias-Fernandez, J., Rovira, C., Davies, G.J., Wilson, K.S., and Cowtan, K.D. (2015). Privateer: software for the conformational validation of carbohydrate structures. Nat. Struct. Mol. Biol. *22*, 833–834.

Andrabi, R., Su, C.Y., Liang, C.H., Shivatare, S.S., Briney, B., Voss, J.E., Nawazi, S.K., Wu, C.Y., Wong, C.H., and Burton, D.R. (2017). Glycans function as anchors for antibodies and help drive HIV broadly neutralizing antibody development. Immunity *47*, 524–537.e3.

Barad, B.A., Echols, N., Wang, R.Y., Cheng, Y., DiMaio, F., Adams, P.D., and Fraser, J.S. (2015). EMRinger: side chain-directed model and map validation for 3D cryo-electron microscopy. Nat. Methods *12*, 943–946.

Behrens, A.J., and Crispin, M. (2017). Structural principles controlling HIV envelope glycosylation. Curr. Opin. Struct. Biol. *44*, 125–133.

Behrens, A.J., Vasiljevic, S., Pritchard, L.K., Harvey, D.J., Andev, R.S., Krumm, S.A., Struwe, W.B., Cupo, A., Kumar, A., Zitzmann, N., et al. (2016). Composition and antigenic effects of individual glycan sites of a trimeric HIV-1 envelope glycoprotein. Cell Rep. *14*, 2695–2706.

Behrens, A.J., Harvey, D.J., Milne, E., Cupo, A., Kumar, A., Zitzmann, N., Struwe, W.B., Moore, J.P., and Crispin, M. (2017a). Molecular architecture of the cleavage-dependent mannose patch on a soluble HIV-1 envelope glycoprotein trimer. J Virol *91*, e01894–01816.

Behrens, A.J., Struwe, W.B., and Crispin, M. (2017b). Glycosylation profiling to evaluate glycoprotein immunogens against HIV-1. Expert Rev. Proteomics *14*, 881–890.

Behrens, A.J., Kumar, A., Medina-Ramirez, M., Cupo, A., Marshall, K., Cruz Portillo, V.M., Harvey, D.J., Ozorowski, G., Zitzmann, N., Wilson, I.A., et al. (2018). Integrity of glycosylation processing of a glycan-depleted trimeric HIV-1 immunogen targeting key B-cell lineages. J. Proteome Res. *17*, 987–999.

Blattner, C., Lee, J.H., Sliepen, K., Derking, R., Falkowska, E., de la Pena, A.T., Cupo, A., Julien, J.P., van Gils, M., Lee, P.S., et al. (2014). Structural delineation of a quaternary, cleavage-dependent epitope at the gp41-gp120 interface on intact HIV-1 Env trimers. Immunity *40*, 669–680.

Bonomelli, C., Doores, K.J., Dunlop, D.C., Thaney, V., Dwek, R.A., Burton, D.R., Crispin, M., and Scanlan, C.N. (2011). The glycan shield of HIV is predominantly oligomannose independently of production system or viral clade. PLoS One *6*, e23521.

Burton, D.R. (2017). What are the most powerful immunogen design vaccine strategies? Reverse vaccinology 2.0 shows great promise. Cold Spring Harb. Perspect. Biol. *9*, a030262.

Burton, D.R., Poignard, P., Stanfield, R.L., and Wilson, I.A. (2012). Broadly neutralizing antibodies present new prospects to counter highly antigenically diverse viruses. Science *337*, 183–186.

Calarese, D.A., Scanlan, C.N., Zwick, M.B., Deechongkit, S., Mimura, Y., Kunert, R., Zhu, P., Wormald, M.R., Stanfield, R.L., Roux, K.H., et al. (2003). Antibody domain exchange is an immunological solution to carbohydrate cluster recognition. Science *300*, 2065–2071.

Calarese, D.A., Lee, H.K., Huang, C.Y., Best, M.D., Astronomo, R.D., Stanfield, R.L., Katinger, H., Burton, D.R., Wong, C.H., and Wilson, I.A. (2005). Dissection of the carbohydrate specificity of the broadly neutralizing anti-HIV-1 antibody 2G12. Proc. Natl. Acad. Sci. U S A *102*, 13372–13377.

Cao, L., Diedrich, J.K., Kulp, D.W., Pauthner, M., He, L., Park, S.R., Sok, D., Su, C.Y., Delahunty, C.M., Menis, S., et al. (2017). Global site-specific N-glycosylation analysis of HIV envelope glycoprotein. Nat. Commun. *8*, 14954.

Cao, L., Pauthner, M., Andrabi, R., Rantalainen, K., Berndsen, Z., Diedrich, J.K., Menis, S., Sok, D., Bastidas, R., Park, S.R., et al. (2018). Differential processing of HIV envelope glycans on the virus and soluble recombinant trimer. Nat. Commun. *9*, 3693.

Carragher, B., Kisseberth, N., Kriegman, D., Milligan, R.A., Potter, C.S., Pulokas, J., and Reilein, A. (2000). Leginon: an automated system for acquisition of images from vitreous ice specimens. J. Struct. Biol. *132*, 33–45.

Chuang, G.Y., Zhou, J., Acharya, P., Rawi, R., Shen, C.H., Sheng, Z., Zhang, B., Zhou, T., Bailer, R.T., Dandey, V.P., et al. (2019). Structural survey of broadly neutralizing antibodies targeting the HIV-1 Env trimer delineates epitope categories and characteristics of recognition. Structure *27*, 196–206.e6.

Coss, K.P., Vasiljevic, S., Pritchard, L.K., Krumm, S.A., Glaze, M., Madzorera, S., Moore, P.L., Crispin, M., and Doores, K.J. (2016). HIV-1 glycan density drives the persistence of the mannose patch within an infected individual. J. Virol. *90*, 11132–11144.

Crispin, M., Ward, A.B., and Wilson, I.A. (2018). Structure and immune recognition of the HIV glycan shield. Annu. Rev. Biophys. *47*, 499–523.

Crooks, E.T., Tong, T., Chakrabarti, B., Narayan, K., Georgiev, I.S., Menis, S., Huang, X., Kulp, D., Osawa, K., Muranaka, J., et al. (2015). Vaccine-elicited tier 2 HIV-1 neutralizing antibodies bind to quaternary epitopes involving glycan-deficient patches proximal to the CD4 binding site. PLoS Pathog. *11*, e1004932.

Crooks, E.T., Osawa, K., Tong, T., Grimley, S.L., Dai, Y.D., Whalen, R.G., Kulp, D.W., Menis, S., Schief, W.R., and Binley, J.M. (2017). Effects of partially dismantling the CD4 binding site glycan fence of HIV-1 envelope glycoprotein trimers on neutralizing antibody induction. Virology *505*, 193–209.

Dacheux, L., Moreau, A., Ataman-Onal, Y., Biron, F., Verrier, B., and Barin, F. (2004). Evolutionary dynamics of the glycan shield of the human immunodeficiency virus envelope during natural infection and implications for exposure of the 2G12 epitope. J. Virol. *78*, 12625–12637.

Dey, A.K., Cupo, A., Ozorowski, G., Sharma, V.K., Behrens, A.J., Go, E.P., Ketas, T.J., Yasmeen, A., Klasse, P.J., Sayeed, E., et al. (2018). cGMP production and analysis of BG505 SOSIP.664, an extensively glycosylated, trimeric HIV-1 envelope glycoprotein vaccine candidate. Biotechnol. Bioeng. *115*, 885–899.

DiMaio, F., Song, Y., Li, X., Brunner, M.J., Xu, C., Conticello, V., Egelman, E., Marlovits, T., Cheng, Y., and Baker, D. (2015). Atomic-accuracy models from 4.5-Å cryo-electron microscopy data with density-guided iterative local refinement. Nat. Methods *12*, 361–365.

Doores, K.J., and Burton, D.R. (2010). Variable loop glycan dependency of the broad and potent HIV-1-neutralizing antibodies PG9 and PG16. J. Virol. *84*, 10510–10521.

Doores, K.J., Bonomelli, C., Harvey, D.J., Vasiljevic, S., Dwek, R.A., Burton, D.R., Crispin, M., and Scanlan, C.N. (2010a). Envelope glycans of immunodeficiency virions are almost entirely oligomannose antigens. Proc. Natl. Acad. Sci. U S A *107*, 13800–13805.

Doores, K.J., Fulton, Z., Huber, M., Wilson, I.A., and Burton, D.R. (2010b). Antibody 2G12 recognizes di-mannose equivalently in domain- and nondomain-exchanged forms but only binds the HIV-1 glycan shield if domain exchanged. J. Virol. *84*, 10690–10699.

Doores, K.J., Huber, M., Le, K.M., Wang, S.K., Doyle-Cooper, C., Cooper, A., Pantophlet, R., Wong, C.H., Nemazee, D., and Burton, D.R. (2013). 2G12-expressing B cell lines may aid in HIV carbohydrate vaccine design strategies. J. Virol. *87*, 2234–2241.

Dunlop, D.C., Bonomelli, C., Mansab, F., Vasiljevic, S., Doores, K.J., Wormald, M.R., Palma, A.S., Feizi, T., Harvey, D.J., Dwek, R.A., et al. (2010). Polysaccharide mimicry of the epitope of the broadly neutralizing anti-HIV antibody, 2G12, induces enhanced antibody responses to self oligomannose glycans. Glycobiology *20*, 812–823.

Emsley, P., and Crispin, M. (2018). Structural analysis of glycoproteins: building N-linked glycans with Coot. Acta Crystallogr. D Struct. Biol. *74*, 256–263.

Falkowska, E., Le, K.M., Ramos, A., Doores, K.J., Lee, J.H., Blattner, C., Ramirez, A., Derking, R., van Gils, M.J., Liang, C.H., et al. (2014). Broadly neutralizing HIV antibodies define a glycan-dependent epitope on the prefusion conformation of gp41 on cleaved envelope trimers. Immunity *40*, 657–668.

Frenz, B., Ramisch, S., Borst, A.J., Walls, A.C., Adolf-Bryfogle, J., Schief, W.R., Veesler, D., and DiMaio, F. (2019). automatically fixing errors in glycoprotein structures with Rosetta. Structure *27*, 134–139.e3.

Gach, J.S., Mara, K.J.V., LaBranche, C.C., van Gils, M.J., McCoy, L.E., Klasse, P.J., Montefiori, D.C., Sanders, R.W., Moore, J.P., and Forthal, D.N. (2019). Antibody responses elicited by immunization with BG505 trimer immune complexes. J. Virol. *93*, e01188–19.

van Gils, M.J., and Sanders, R.W. (2014). In vivo protection by broadly neutralizing HIV antibodies. Trends Microbiol. *22*, 550–551.

van Gils, M.J., Euler, Z., Schweighardt, B., Wrin, T., and Schuitemaker, H. (2009). Prevalence of cross-reactive HIV-1-neutralizing activity in HIV-1-infected patients with rapid or slow disease progression. AIDS *23*, 2405–2414.

Go, E.P., Liao, H.X., Alam, S.M., Hua, D., Haynes, B.F., and Desaire, H. (2013). Characterization of host-cell line specific glycosylation profiles of early transmitted/founder HIV-1 gp120 envelope proteins. J. Proteome Res. *12*, 1223–1234.

Gristick, H.B., von Boehmer, L., West, A.P., Jr., Schamber, M., Gazumyan, A., Golijanin, J., Seaman, M.S., Fatkenheuer, G., Klein, F., Nussenzweig, M.C., et al. (2016). Natively glycosylated HIV-1 Env structure reveals new mode for antibody recognition of the CD4-binding site. Nat. Struct. Mol. Biol. *23*, 906–915.

Hoot, S., McGuire, A.T., Cohen, K.W., Strong, R.K., Hangartner, L., Klein, F., Diskin, R., Scheid, J.F., Sather, D.N., Burton, D.R., et al. (2013). Recombinant HIV envelope proteins fail to engage germline versions of anti-CD4bs bNAbs. PLoS Pathog. *9*, e1003106.

Huang, J., Kang, B.H., Pancera, M., Lee, J.H., Tong, T., Feng, Y., Imamichi, H., Georgiev, I.S., Chuang, G.Y., Druz, A., et al. (2014). Broad and potent HIV-1 neutralization by a human antibody that binds the gp41-gp120 interface. Nature *515*, 138–142.

Jardine, J., Julien, J.P., Menis, S., Ota, T., Kalyuzhniy, O., McGuire, A., Sok, D., Huang, P.S., MacPherson, S., Jones, M., et al. (2013). Rational HIV immunogen design to target specific germline B cell receptors. Science *340*, 711–716.

Klasse, P.J., LaBranche, C.C., Ketas, T.J., Ozorowski, G., Cupo, A., Pugach, P., Ringe, R.P., Golabek, M., van Gils, M.J., Guttman, M., et al. (2016). Sequential and simultaneous immunization of rabbits with HIV-1 envelope glycoprotein SOSIP.664 trimers from clades A, B and C. PLoS Pathog. *12*, e1005864.

Klasse, P.J., Ketas, T.J., Cottrell, C.A., Ozorowski, G., Debnath, G., Camara, D., Francomano, E., Pugach, P., Ringe, R.P., LaBranche, C.C., et al. (2018). Epitopes for neutralizing antibodies induced by HIV-1 envelope glycoprotein BG505 SOSIP trimers in rabbits and macaques. PLoS Pathog. *14*, e1006913.

Kong, L., Lee, J.H., Doores, K.J., Murin, C.D., Julien, J.P., McBride, R., Liu, Y., Marozsan, A., Cupo, A., Klasse, P.J., et al. (2013). Supersite of immune vulnerability on the glycosylated face of HIV-1 envelope glycoprotein gp120. Nat. Struct. Mol. Biol. *20*, 796–803.

Kong, L., Wilson, I.A., and Kwong, P.D. (2015). Crystal structure of a fully glycosylated HIV-1 gp120 core reveals a stabilizing role for the glycan at Asn262. Proteins *83*, 590–596.

Krissinel, E. (2015). Stock-based detection of protein oligomeric states in jsPISA. Nucleic Acids Res. *43*, W314–W319.

Lee, J.H., de Val, N., Lyumkis, D., and Ward, A.B. (2015). Model building and refinement of a natively glycosylated HIV-1 Env protein by high-resolution cryoelectron microscopy. Structure *23*, 1943–1951.

Lemmin, T., Soto, C., Stuckey, J., and Kwong, P.D. (2017). Microsecond dynamics and network analysis of the HIV-1 SOSIP Env trimer reveal collective behavior and conserved microdomains of the glycan shield. Structure *25*, 1631–1639.e2.

Ludtke, S.J., Baldwin, P.R., and Chiu, W. (1999). EMAN: semiautomated software for high-resolution single-particle reconstructions. J. Struct. Biol. *128*, 82–97.

Lutteke, T., Frank, M., and von der Lieth, C.W. (2005). Carbohydrate Structure Suite (CSS): analysis of carbohydrate 3D structures derived from the PDB. Nucleic Acids Res. *33*, D242–D246.

Ma, B.J., Alam, S.M., Go, E.P., Lu, X., Desaire, H., Tomaras, G.D., Bowman, C., Sutherland, L.L., Scearce, R.M., Santra, S., et al. (2011). Envelope deglycosylation enhances antigenicity of HIV-1 gp41 epitopes for both broad neutralizing antibodies and their unmutated ancestor antibodies. PLoS Pathog. *7*, e1002200.

McCoy, L.E., Falkowska, E., Doores, K.J., Le, K., Sok, D., van Gils, M.J., Euler, Z., Burger, J.A., Seaman, M.S., Sanders, R.W., et al. (2015). Incomplete neutralization and deviation from sigmoidal neutralization curves for HIV broadly neutralizing monoclonal antibodies. PLoS Pathog. *11*, e1005110.

McCoy, L.E., van Gils, M.J., Ozorowski, G., Messmer, T., Briney, B., Voss, J.E., Kulp, D.W., Macauley, M.S., Sok, D., Pauthner, M., et al. (2016). Holes in the

glycan shield of the native HIV envelope are a target of trimer-elicited neutralizing antibodies. Cell Rep. *16*, 2327–2338.

McGuire, A.T., Hoot, S., Dreyer, A.M., Lippy, A., Stuart, A., Cohen, K.W., Jardine, J., Menis, S., Scheid, J.F., West, A.P., et al. (2013). Engineering HIV envelope protein to activate germline B cell receptors of broadly neutralizing anti-CD4 binding site antibodies. J. Exp. Med. *210*, 655–663.

McGuire, A.T., Glenn, J.A., Lippy, A., and Stamatatos, L. (2014). Diverse recombinant HIV-1 Envs fail to activate B cells expressing the germline B cell receptors of the broadly neutralizing anti-HIV-1 antibodies PG9 and 447-52D. J. Virol. *88*, 2645–2657.

McLellan, J.S., Pancera, M., Carrico, C., Gorman, J., Julien, J.P., Khayat, R., Louder, R., Pejchal, R., Sastry, M., Dai, K., et al. (2011). Structure of HIV-1 gp120 V1/V2 domain with broadly neutralizing antibody PG9. Nature *480*, 336–343.

Medina-Ramirez, M., Garces, F., Escolano, A., Skog, P., de Taeye, S.W., Del Moral-Sanchez, I., McGuire, A.T., Yasmeen, A., Behrens, A.J., Ozorowski, G., et al. (2017). Design and crystal structure of a native-like HIV-1 envelope trimer that engages multiple broadly neutralizing antibody precursors in vivo. J. Exp. Med. *214*, 2573–2590.

Moore, P.L., Gray, E.S., Wibmer, C.K., Bhiman, J.N., Nonyane, M., Sheward, D.J., Hermanus, T., Bajimaya, S., Tumba, N.L., Abrahams, M.R., et al. (2012). Evolution of an HIV glycan-dependent broadly neutralizing antibody epitope through immune escape. Nat. Med. *18*, 1688–1692.

Murin, C.D., Julien, J.P., Sok, D., Stanfield, R.L., Khayat, R., Cupo, A., Moore, J.P., Burton, D.R., Wilson, I.A., and Ward, A.B. (2014). Structure of 2G12 Fab2 in complex with soluble and fully glycosylated HIV-1 Env by negative-stain single-particle electron microscopy. J. Virol. *88*, 10177–10188.

Pancera, M., Shahzad-Ul-Hussan, S., Doria-Rose, N.A., McLellan, J.S., Bailer, R.T., Dai, K., Loesgen, S., Louder, M.K., Staupe, R.P., Yang, Y., et al. (2013). Structural basis for diverse N-glycan recognition by HIV-1-neutralizing V1-V2-directed antibody PG16. Nat. Struct. Mol. Biol. *20*, 804–813.

Pegu, A., Hessell, A.J., Mascola, J.R., and Haigwood, N.L. (2017). Use of broadly neutralizing antibodies for HIV-1 prevention. Immunol. Rev. *275*, 296–312.

Pejchal, R., Doores, K.J., Walker, L.M., Khayat, R., Huang, P.S., Wang, S.K., Stanfield, R.L., Julien, J.P., Ramos, A., Crispin, M., et al. (2011). A potent and broad neutralizing antibody recognizes and penetrates the HIV glycan shield. Science *334*, 1097–1103.

Pettersen, E.F., Goddard, T.D., Huang, C.C., Couch, G.S., Greenblatt, D.M., Meng, E.C., and Ferrin, T.E. (2004). UCSF Chimera—a visualization system for exploratory research and analysis. J. Comput. Chem. *25*, 1605–1612.

Posner, M.R., Hideshima, T., Cannon, T., Mukherjee, M., Mayer, K.H., and Byrn, R.A. (1991). An IgG human monoclonal antibody that reacts with HIV-1/GP120, inhibits virus binding to cells, and neutralizes infection. J. Immunol. *146*, 4325–4332.

Pritchard, L.K., Spencer, D.I., Royle, L., Bonomelli, C., Seabright, G.E., Behrens, A.J., Kulp, D.W., Menis, S., Krumm, S.A., Dunlop, D.C., et al. (2015a). Glycan clustering stabilizes the mannose patch of HIV-1 and preserves vulnerability to broadly neutralizing antibodies. Nat. Commun. *6*, 7479.

Pritchard, L.K., Spencer, D.I., Royle, L., Vasiljevic, S., Krumm, S.A., Doores, K.J., and Crispin, M. (2015b). Glycan microheterogeneity at the PGT135 antibody recognition site on HIV-1 gp120 reveals a molecular mechanism for neutralization resistance. J. Virol. *89*, 6952–6959.

Pritchard, L.K., Vasiljevic, S., Ozorowski, G., Seabright, G.E., Cupo, A., Ringe, R., Kim, H.J., Sanders, R.W., Doores, K.J., Burton, D.R., et al. (2015c). Structural constraints determine the glycosylation of HIV-1 envelope trimers. Cell Rep. *11*, 1604–1613.

Punjani, A., Rubinstein, J.L., Fleet, D.J., and Brubaker, M.A. (2017). cryoSPARC: algorithms for rapid unsupervised cryo-EM structure determination. Nat. Methods *14*, 290–296.

Ringe, R.P., Pugach, P., Cottrell, C.A., LaBranche, C.C., Seabright, G.E., Ketas, T.J., Ozorowski, G., Kumar, S., Schorcht, A., van Gils, M.J., et al. (2019). Closing and opening holes in the glycan shield of HIV-1 envelope glycoprotein SOSIP trimers can redirect the neutralizing antibody response to the newly unmasked epitopes. J. Virol. *93*, e01656–18.

Robinson, J.E., Holton, D., Pacheco-Morell, S., Liu, J., and McMurdo, H. (1990). Identification of conserved and variant epitopes of human immunodeficiency virus type 1 (HIV-1) gp120 by human monoclonal antibodies produced by EBV-transformed cell lines. AIDS Res. Hum. Retroviruses *6*, 567–579.

Sanders, R.W., and Moore, J.P. (2017). Native-like Env trimers as a platform for HIV-1 vaccine design. Immunol. Rev. *275*, 161–182.

Sanders, R.W., Venturi, M., Schiffner, L., Kalyanaraman, R., Katinger, H., Lloyd, K.O., Kwong, P.D., and Moore, J.P. (2002). The mannose-dependent epitope for neutralizing antibody 2G12 on human immunodeficiency virus type 1 glycoprotein gp120. J. Virol. *76*, 7293–7305.

Sanders, R.W., van Anken, E., Nabatov, A.A., Liscaljet, I.M., Bontjer, I., Eggink, D., Melchers, M., Busser, E., Dankers, M.M., Groot, F., et al. (2008). The carbohydrate at asparagine 386 on HIV-1 gp120 is not essential for protein folding and function but is involved in immune evasion. Retrovirology *5*, 10.

Sanders, R.W., Derking, R., Cupo, A., Julien, J.P., Yasmeen, A., de Val, N., Kim, H.J., Blattner, C., de la Pena, A.T., Korzun, J., et al. (2013). A next-generation cleaved, soluble HIV-1 Env trimer, BG505 SOSIP.664 gp140, expresses multiple epitopes for broadly neutralizing but not non-neutralizing antibodies. PLoS Pathog. *9*, e1003618.

Scanlan, C.N., Pantophlet, R., Wormald, M.R., Ollmann Saphire, E., Stanfield, R., Wilson, I.A., Katinger, H., Dwek, R.A., Rudd, P.M., and Burton, D.R. (2002). The broadly neutralizing anti-human immunodeficiency virus type 1 antibody 2G12 recognizes a cluster of alpha1→2 mannose residues on the outer face of gp120. J. Virol. *76*, 7306–7321.

Scanlan, C.N., Offer, J., Zitzmann, N., and Dwek, R.A. (2007). Exploiting the defensive sugars of HIV-1 for drug and vaccine design. Nature *446*, 1038–1045.

Scharf, L., Scheid, J.F., Lee, J.H., West, A.P., Jr., Chen, C., Gao, H., Gnanapragasam, P.N., Mares, R., Seaman, M.S., Ward, A.B., et al. (2014). Antibody 8ANC195 reveals a site of broad vulnerability on the HIV-1 envelope spike. Cell Rep. *7*, 785–795.

Simek, M.D., Rida, W., Priddy, F.H., Pung, P., Carrow, E., Laufer, D.S., Lehrman, J.K., Boaz, M., Tarragona-Fiol, T., Miiro, G., et al. (2009). Human immunodeficiency virus type 1 elite neutralizers: individuals with broad and potent neutralizing activity identified by using a high-throughput neutralization assay together with an analytical selection algorithm. J. Virol. *83*, 7337–7348.

Stamatatos, L., Pancera, M., and McGuire, A.T. (2017). Germline-targeting immunogens. Immunol. Rev. *275*, 203–216.

Steichen, J.M., Kulp, D.W., Tokatlian, T., Escolano, A., Dosenovic, P., Stanfield, R.L., McCoy, L.E., Ozorowski, G., Hu, X., Kalyuzhniy, O., et al. (2016). HIV vaccine design to target germline precursors of glycan-dependent broadly neutralizing antibodies. Immunity *45*, 483–496.

Stephenson, K.E., and Barouch, D.H. (2016). Broadly neutralizing antibodies for HIV eradication. Curr. HIV/AIDS Rep. *13*, 31–37.

Stewart-Jones, G.B., Soto, C., Lemmin, T., Chuang, G.Y., Druz, A., Kong, R., Thomas, P.V., Wagh, K., Zhou, T., Behrens, A.J., et al. (2016). Trimeric HIV-1-Env structures define glycan shields from clades A, B, and G. Cell *165*, 813–826.

Struwe, W.B., Chertova, E., Allen, J.D., Seabright, G.E., Watanabe, Y., Harvey, D.J., Medina-Ramirez, M., Roser, J.D., Smith, R., Westcott, D., et al. (2018). Site-specific glycosylation of virion-derived HIV-1 Env is mimicked by a soluble trimeric immunogen. Cell Rep. *24*, 1958–1966.e5.

de Taeye, S.W., Ozorowski, G., Torrents de la Pena, A., Guttman, M., Julien, J.P., van den Kerkhof, T.L., Burger, J.A., Pritchard, L.K., Pugach, P., Yasmeen, A., et al. (2015). Immunogenicity of stabilized HIV-1 envelope trimers with reduced exposure of non-neutralizing epitopes. Cell *163*, 1702–1715.

Torrents de la Pena, A., Julien, J.P., de Taeye, S.W., Garces, F., Guttman, M., Ozorowski, G., Pritchard, L.K., Behrens, A.J., Go, E.P., Burger, J.A., et al. (2017). Improving the immunogenicity of native-like HIV-1 envelope trimers by hyperstabilization. Cell Rep. *20*, 1805–1817.

CellPress
OPEN ACCESS

Trkola, A., Purtscher, M., Muster, T., Ballaun, C., Buchacher, A., Sullivan, N., Srinivasan, K., Sodroski, J., Moore, J.P., and Katinger, H. (1996). Human monoclonal antibody 2G12 defines a distinctive neutralization epitope on the gp120 glycoprotein of human immunodeficiency virus type 1. J. Virol. *70*, 1100–1108.

Voss, J.E., Andrabi, R., McCoy, L.E., de Val, N., Fuller, R.P., Messmer, T., Su, C.Y., Sok, D., Khan, S.N., Garces, F., et al. (2017). Elicitation of neutralizing antibodies targeting the V2 apex of the HIV envelope trimer in a wild-type animal model. Cell Rep. *21*, 222–235.

Wagh, K., Kreider, E.F., Li, Y., Barbian, H.J., Learn, G.H., Giorgi, E., Hraber, P.T., Decker, T.G., Smith, A.G., Gondim, M.V., et al. (2018). Completeness of HIV-1 envelope glycan shield at transmission determines neutralization breadth. Cell Rep. *25*, 893–908.e7.

Walker, L.M., Phogat, S.K., Chan-Hui, P.Y., Wagner, D., Phung, P., Goss, J.L., Wrin, T., Simek, M.D., Fling, S., Mitcham, J.L., et al. (2009). Broad and potent neutralizing antibodies from an African donor reveal a new HIV-1 vaccine target. Science *326*, 285–289.

Walker, L.M., Huber, M., Doores, K.J., Falkowska, E., Pejchal, R., Julien, J.P., Wang, S.K., Ramos, A., Chan-Hui, P.Y., Moyle, M., et al. (2011). Broad neutralization coverage of HIV by multiple highly potent antibodies. Nature *477*, 466–470.

Wang, W., Nie, J., Prochnow, C., Truong, C., Jia, Z., Wang, S., Chen, X.S., and Wang, Y. (2013). A systematic study of the N-glycosylation sites of HIV-1 envelope protein on infectivity and antibody-mediated neutralization. Retrovirology *10*, 14.

Ward, A.B., and Wilson, I.A. (2017). The HIV-1 envelope glycoprotein structure: nailing down a moving target. Immunol. Rev. *275*, 21–32.

Wei, X., Decker, J.M., Wang, S., Hui, H., Kappes, J.C., Wu, X., Salazar-Gonzalez, J.F., Salazar, M.G., Kilby, J.M., Saag, M.S., et al. (2003). Antibody neutralization and escape by HIV-1. Nature *422*, 307–312.

Williams, C.J., Headd, J.J., Moriarty, N.W., Prisant, M.G., Videau, L.L., Deis, L.N., Verma, V., Keedy, D.A., Hintze, B.J., Chen, V.B., et al. (2018). MolProbity: more and better reference data for improved all-atom structure validation. Protein Sci. *27*, 293–315.

Wu, X., Yang, Z.Y., Li, Y., Hogerkorp, C.M., Schief, W.R., Seaman, M.S., Zhou, T., Schmidt, S.D., Wu, L., Xu, L., et al. (2010). Rational design of envelope identifies broadly neutralizing human monoclonal antibodies to HIV-1. Science *329*, 856–861.

Wu, X., Zhou, T., Zhu, J., Zhang, B., Georgiev, I., Wang, C., Chen, X., Longo, N.S., Louder, M., McKee, K., et al. (2011). Focused evolution of HIV-1 neutralizing antibodies revealed by structures and deep sequencing. Science *333*, 1593–1602.

Wyatt, R., and Sodroski, J. (1998). The HIV-1 envelope glycoproteins: fusogens, antigens, and immunogens. Science *280*, 1884–1888.

Xiao, X., Chen, W., Feng, Y., Zhu, Z., Prabakaran, P., Wang, Y., Zhang, M.Y., Longo, N.S., and Dimitrov, D.S. (2009). Germline-like predecessors of broadly neutralizing antibodies lack measurable binding to HIV-1 envelope glycoproteins: implications for evasion of immune responses and design of vaccine immunogens. Biochem. Biophys. Res. Commun. *390*, 404–409.

Zhang, K. (2016). Gctf: real-time CTF determination and correction. J. Struct. Biol. *193*, 1–12.

STAR★METHODS

KEY RESOURCES TABLE

REAGENT or RESOURCE	SOURCE	IDENTIFIER
Antibodies		
PGV04 Fab, 2G12, PG9, PG16, PGT145, PGT151, VRC01, 19b, F105	This paper	N/A
Goat F(ab')2 Anti-Human IgG (Fab')2 (HRP)	Abcam	RRID: ab98535
Chemicals, Peptides, and Recombinant Proteins		
FreeStyle™ MAX Reagent	Thermo Fisher Scientific	Cat# 10259172
Gibco™ OptiPRO™ SFM	Thermo Fisher Scientific	Cat# 10569520
FreeStyle 293F media	Thermo Fisher Scientific	Cat# 12338026
Acetonitrile, 80%, 20% Water with 0.1% Formic Acid, Optima LC/MS	Fisher Scientific	Cat# 15431423
Water with 0.1% Formic Acid (v/v), Optima™ LC/MS Grade	Fisher Scientific	Cat# LS118-212
Acetonitrile	Fisher Scientific	Cat# 10489553
Trifluoroacetic acid	Fisher Scientific	Cat# 10155347
Procainamide hydrochloride	Abcam	Cat# ab120955
$H_2{}^{18}O$	Sigma-Aldrich	Cat# 329878
Dithiothreitol	Sigma-Aldrich	Cat# 43819
Iodacetamide	Sigma-Aldrich	Cat# I1149
Ammonium formate buffer	Waters	Cat# 186007081
Sodium cyanoborohydride	Sigma-Aldrich	Cat# 156159
Dimethyl sulfoxide	Sigma-Aldrich	Cat# D2438
Acetic acid	Fisher Scientific	Cat# 10384970
Peptide-N-glycosidase F	New England Biolabs	Cat# P0705S
Endoglycosidase H	New England Biolabs	Cat# P0702S
Mass spectrometry grade trypsin	Promega	Cat# V5280
Sequencing grade chymotrypsin	Promega	Cat# V1061
Papain	Sigma-Aldrich	Cat# P3125
n-Dodecyl-β-D-maltoside	Anatrace	D310
1-Step™ TMB-Blotting Substrate Solution	Thermo Fisher Scientific	Cat# 34018
Streptavidin (SA) biosensors	Fortébio	Cat# 18-5019
NaCl	Sigma-Aldrich	S7653-1KG
Tris base	Sigma-Aldrich	10708976001
HCl	Sigma-Aldrich	H1758-500ML
$MgCl_2$	Sigma-Aldrich	M8266-1KG
Glycine	Sigma-Aldrich	G7126-1KG
Sodium acetate	Sigma-Aldrich	S2889-1KG
KCl	Sigma-Aldrich	P9333-1KG
PBS	Sigma-Aldrich	P4417-100TAB
Sodium phosphate monobasic monohydrate	Sigma-Aldrich	S9638-1KG
Sodium phosphate dibasic dihydrate	Sigma-Aldrich	71643-1KG
Citric acid monohydrate	Sigma-Aldrich	C1909-1KG
Sodium citrate tribasic dihydrate	Sigma-Aldrich	S4641-1KG
EDTA	Sigma-Aldrich	EDS-500G
L-cysteine	Sigma-Aldrich	W326305-1KG

(*Continued on next page*)

CellPress
OPEN ACCESS

Continued

REAGENT or RESOURCE	SOURCE	IDENTIFIER
Urea	Sigma-Aldrich	U5378-1KG
Critical Commercial Assays		
QuikChange Lightning Site-Directed Mutagenesis kit	Agilent	Cat# 210518
Deposited Data		
Cryo EM map of BG505 SOSIP.664 with 2G12 Fab_2	This paper	EMDB: EMD-20224
Atomic model of BG505 SOSIP.664 with 2G12 Fab_2	This paper	PDB: 6OZC
Cryo-EM structure of PGT128 Fab in complex with BG505 SOSIP.664 Env trimer	(Lee et al., 2015)	PDB: 5ACO
Anti-HIV-1 Fab 2G12 + Man9 re-refinement	(Calarese et al., 2003)	PDB: 6N2X
Crystal Structure of PG9 Fab in Complex with V1V2 Region from HIV-1 strain CAP45	(McLellan et al., 2011)	PDB: 3U4E
Crystal Structure of PG16 Fab in Complex with V1V2 Region from HIV-1 strain ZM109	(Pancera et al., 2013)	PDB: 4DQO
Experimental Models: Cell Lines		
HEK 293F cells	Thermo Fisher Scientific	Cat# R79007
Oligonucleotides		
See Table S5	This paper	N/A
Recombinant DNA		
BG505 SOSIP.664	(Sanders et al., 2013)	N/A
BG505 SOSIP.v5	(Torrents de la Pena et al., 2017)	N/A
BG505 SOSIP.v4.1	(de Taeye et al., 2015)	N/A
BG505 SOSIP.v4.1-2XStrep	This paper	N/A
2G12 light and heavy chains	(Calarese et al., 2003)	N/A
PGT145 light and heavy chains	(Walker et al., 2011)	N/A
PGT151 light and heavy chains	(Blattner et al., 2014)	N/A
PG9 light and heavy chains	(Walker et al., 2011)	N/A
PG16 light and heavy chains	(Walker et al., 2011)	N/A
PGV04 Fab light and heavy chains	(Wu et al., 2011)	N/A
VRC01 light and heavy chains	(Wu et al., 2010)	N/A
19b light and heavy chains	(Robinson et al., 1990)	N/A
F105 light and heavy chains	(Posner et al., 1991)	N/A
Software and Algorithms		
Empower 3.0	Waters	https://www.waters.com/waters/en_GB/Empower-3-Chromatography-Data-Software/nav.htm?cid=513188&locale=en_GB
Masslynx v4.1	Waters	https://www.waters.com/waters/en_GB/MassLynx-MS-Software/nav.htm?locale=en_GB&cid=513662
Driftscope version 2.8	Waters	N/A
ByonicTM (Version 2.7)	Protein Metrics Inc.	https://www.proteinmetrics.com/products/byonic/
ByologicTM software (Version 2.3)	Protein Metrics Inc.	https://www.proteinmetrics.com/products/byologic/
Leginon (version 3.3)	National Resource for Automated Molecular Microscopy (NRAMM)	https://nramm.nysbc.org/software/
cryoSPARC (version 2)	Structura	https://cryosparc.com

(*Continued on next page*)

Continued

REAGENT or RESOURCE	SOURCE	IDENTIFIER
Gctf (version 1.06)	MRC Laboratory of Molecular Biology	https://www.mrc-lmb.cam.ac.uk/kzhang/Gctf/
UCSF Chimera (version 1.13)	UCSF	https://www.cgl.ucsf.edu/chimera/download.html
RosettaRelax (version 3.10)	University of Washington	https://www.rosettacommons.org/software
jsPISA (version 2.0.4)	Collaborative Computational Project No. 4 (CCP4)	http://www.ccp4.ac.uk/pisa/
Rosetta (version 3.10)	University of Washington	https://www.rosettacommons.org/software
Molprobity (version 4.4)	Duke University	http://molprobity.biochem.duke.edu
EMRinger	UCSF (Barad et al., 2015)	N/A
Privateer	Collaborative Computational Project No. 4 (CCP4)	http://www.ccp4.ac.uk/html/privateer.html
CArbohydrate Ramachandran Plot (CARP)	Glycosciences.de	http://www.glycosciences.de/tools/carp/
pdb-care	Glycosciences.de	http://www.glycosciences.de/tools/pdb-care/
Coot (version 0.9-pre)	MRC Laboratory of Molecular Biology	https://www2.mrc-lmb.cam.ac.uk/personal/pemsley/coot/
Octet Data Analysis software	Fortébio	https://www.fortebio.com/products/octet-systems-software
Other		
HiTrap KappaSelect column	GE Healthcare	Cat# 17545812
Mono-S column	GE Healthcare	Cat# 17516801
SnakeSkin™ 3.5K MWCO	Thermo Fisher Scientific	Cat# 68035
Superose 6i column	GE Healthcare	Cat# 29091596
HiTrap Protein A HP column	GE Healthcare	Cat# 17040301
Superdex 200 10/300 GL column	GE Healthcare	Cat# 17517501
Econo-Column® Chromatography Columns	Bio-Rad	Cat# 7371512
CNBr-activated Sepharose 4B beads	GE Healthcare	Cat# 17043001
Glycan BEH Amide column (2.1 mm x 100 mm, 1.7 μM)	Waters	Cat# 186004741
EasySpray PepMap RSLC C18 column (75 μm x 75 cm)	Thermo Fisher Scientific	Cat# ES805
PVDF protein-binding membrane	Millipore	Cat# MAIPS4510
C18 ZipTip	Merck Milipore	Cat# ZTC18S008
Spe-ed Amide 2 cartridges	Applied Separations	Cat# 4821
Corning® 96 Well EIA/RIA Assay Microplate	Merck Millipore	Cat# CLS3590
Vivaspin 500, 3 kDa MWCO, Polyethersulfone	Sigma-Aldrich	Cat# GE28-9322-18
Amicon® Ultra, 100 MWCO concentrator	Merck Millipore	UFC910024
Amicon® Ultra, 10 MWCO concentrator	Merck Millipore	UFC901024
Vivaspin 20, 100kDa MWCO	Sigma-Aldrich	Cat# Z614661
C-Flat grid	Protochips, Inc	CF-2/2-4C
Stericup-GP Sterile Vacuum Filtration System	Merck Millipore	SCGPU02RE
Solarus Advanced Plasma Cleaning System	Gatan	Model# 950
SDS-PAGE 4-20% Tris-glycine gel	Invitrogen	Cat# XP04205BOX
Nafion 117 membrane	Sigma-Aldrich	Cat# 274674-1EA

RESOURCE AVAILABILITY

Lead Contact

Further information and requests for resources and reagents should be directed to and will be fulfilled by the Lead Contact, Max Crispin (max.crispin@soton.ac.uk).

Materials Availability

This study did not generate new unique reagents.

Data and Code Availability

The cryo-EM map and atomic model of BG505 SOSIP.664 with 2G12 Fab_2 was deposited with the Electron Microscopy Data Bank and the Protein Data Bank under accession codes EMD-20224 and 6OZC.

EXPERIMENTAL MODEL AND SUBJECT DETAILS

HEK 293F Cell Culture

HEK 293F cells (female) were maintained in FreeStyle 293F media at a density of 0.1-3x10^6 cells per mL at 37°C and 125 rpm shaking. The cells were transfected with FreeStyle™ MAX Reagent and Gibco™ OptiPRO™ SFM at a density of 1x10^6 cells per mL and incubated for 5 days at 37°C with 8% CO_2 and 125 rpm shaking.

METHOD DETAILS

Glycan Site Mutagenesis

Asparagine residues within the N295, N339, N363, N386, N392, N411 and N448 consensus sequence, N-X-S/T (where X $\neq$ P), were mutated to alanine or glutamine. For the N332 site, we reverted the asparagine to threonine, as per the parental BG505 sequence. To introduce N-linked glycan sites at the N241 and N289 sites, the serine at the 241 position was mutated to asparagine, and/or the proline at the 291 position was mutated to serine. Mutants were created using the QuikChange Lightning site-directed mutagenesis kit (Agilent). Briefly, 5 μl of reaction buffer, 10-100 ng of BG505 SOSIP template DNA, 125 ng of each oligonucleotide primer, 1 μl of dNTP mix, 1.5 μl of QuikSolution reagent, 1 μl of QuikChange Lightning Enzyme and H_2O to a final volume of 50 μl were incubated for 2 minutes at 95°C, 18 cycles of 20 seconds at 95°C, 10 seconds at 60°C, and 4 minutes at 68°C, and a further 5 minutes at 68°C in a Thermal Cycler (Bio-Rad), prior to transformation into competent cells. The primers used are given in Table S5.

EXPRESSION AND PURIFICATION OF PROTEINS

BG505 SOSIP.664, SOSIP.v5, SOSIP.v4.1, and SOSIP.v4.1-2xStrep proteins were transiently (co-)expressed in HEK 293F cells with a Furin expression plasmid at a ratio of (4:1). BG505 SOSIP proteins were purified using either 2G12, PGT145, or PGT151 affinity chromatography, as previously described (Sanders et al., 2013). Briefly, transfection supernatants were vacuum filtered through 0.2 μm filters (Merck) and then passed (0.5–1 mL/min flow rate) over the column. The columns (Econo-Column Chromatography Columns, Bio-Rad) were made from CNBr-activated Sepharose 4B beads (GE Healthcare) coupled to the bnAb. A 0.5 M NaCl, 20 mM Tris, pH 8.0 buffer was used for column equilibration and washing. Bound Env proteins were eluted using 3 M $MgCl_2$. The eluted proteins were immediately buffer exchanged into 75 mM NaCl, 10 mM Tris, pH 8.0. Comparisons were only drawn between BG505 SOSIP proteins purified using the same antibody.

PGV04 Fab and 2G12, PGT145, PGT151, PG16, PG9, VRC01, 19b, and F105 IgG were produced by co-expression of the heavy and light chain genes in HEK 293F cells. PGV04 Fab was purified using KappaSelect HiTrap affinity column (GE Healthcare) and eluted in 1.4 mL fractions using 0.1 M glycine, pH 3.0 into wells contain 0.5 mL 1 M Tris, pH 9.0. The KappaSelect elution was dialyzed against 20 mM sodium acetate, pH 5.6 at 4°C overnight using SnakeSkin™ 3.5K MWCO dialysis tubing (ThermoFisher). The PGV04 Fab was then loaded onto a Mono S cation exchange column (GE Healthcare) and eluted using a gradient of 20 mM sodium acetate, pH 5.6, with 1 M KCl. Fractions containing the PGV04 Fab were pooled, concentrated, and buffer exchanged into PBS using a 10 kDa concentrator (Amicon Ultra, Millipore). Full length IgG was purified with a Protein A column (GE Healthcare). Column equilibration and washing was carried out with a 20 mM $NaPO_4$, pH 7.5 buffer, and 0.1 M citric acid, pH 3.0 was used for elution. 2G12 Fab_2 was prepared by digesting 2G12 IgG with with 2% (w/w) activated papain in 100 mM Tris, 2 mM EDTA, 10 mM L-cysteine, pH 8.0 at 37°C for 3 hours. The digestion reaction was quenched with iodoacetamide to a final concentration of 30 mM passed over Protein A HiTrap affinity column (GE Healthcare) to capture Fc and undigested IgG. The flow through containing the 2G12 Fab_2 was further purified using a Superdex 200 10/300 GL column (GE Healthcare) preequilibrated with TBS pH 7.4 (Calarese et al., 2003).

Cryo-Electron Microscopy and Model Building

BG505 SOSIP.664 purified by 2G12 affinity chromatography was mixed with 10-fold molar excess PGV04 Fab and 2G12 Fab_2 and incubated overnight at room temperature. The trimer/Fab complex was purified by size exclusion chromatography using a Superose 6 column (GE Healthcare). The fractions containing the complex were pooled and concentrated using a 100 kDa concentrator

(Amicon Ultra, Millipore) to ~40 μL at 2.5 mg/mL. 5 μL of the complex was incubated with 3 μL of a fresh *n*-dodecyl-β-D-maltoside solution at 1.8 mM. A 3 μL aliquot of the complex/detergent mix was applied to a C-Flat grid (CF-2/2-4C, Electron Microscopy Sciences, Protochips, Inc.) which had been plasma cleaned for 5 seconds using a mixture of Ar/O_2 (Gatan Solarus 950 Plasma system). The sample was manually blotted off, and then immediately plunged into liquid ethane using a manual freeze plunger.

Movies were collected via the Leginon interface on a FEI Titan Krios operating at 300 keV mounted with a Gatan K2 direct electron detector (Carragher et al., 2000). Each movie was collected in counting mode at 22,500 × nominal magnification resulting in a calibrated pixel size of 1.31 Å/pix at the object level. A dose rate of ~10 e^-/(pix*s) was used; exposure time was 200 ms per frame. The data collection resulted in a total of 2,184 movies containing 50 frames each. Total dose per movie was 76 $e^-/Å^2$. Data were collected with a defocus range of -1.5 to -3.0 microns. Movies were imported into cryoSPARC v2 and frames were aligned using full-frame motion correction (Punjani et al., 2017). The contrast transfer function (CTF) for each aligned micrograph was estimated using Gctf (Zhang, 2016). The HIV Env portion of PDB 5ACO was converted to an EM density and low pass filtered to 40 Å using pdb2mrc and subsequently used as a template for particle picking within cryoSPARC v2 (Lee et al., 2015; Ludtke et al., 1999; Punjani et al., 2017). 2D classification, Ab-initio 3D reconstruction, homogenous 3D refinement, and local motion correction were conducted with cryoSPARC v2 (Punjani et al., 2017). Per-particle CTF estimation was conducted using using Gctf (Zhang, 2016). The final C3 symmetric, 3.8 Å reconstruction was obtained using non-uniform refinement within cryoSPARC v2 (Punjani et al., 2017). The per gp120 protomer occupancy of the PGV04 Fab was low and resulted in weak density when the EM data were refined using C3 symmetry. As such, the PGV04 Fab was not included during the model building process.

An initial model was made by docking the gp120 and gp41 domains from the BG505 SOSIP.664 structure (PDB 5ACO) and the 2G12 Fab_2 structure (PDB 6N2X) into the EM density map using UCSF Chimera (Calarese et al., 2003; Lee et al., 2015; Pettersen et al., 2004). The resulting model was symmetrically refined into the EM density map using RosettaRelax (DiMaio et al., 2015). Glycans were built manually using the Carbohydrate module in Coot and refined into the EM density map using Rosetta (Emsley and Crispin, 2018; Frenz et al., 2019). Model accuracy and fit-to-map were assessed using Molprobity, EMRinger, Privateer, CARP, and pdb-care (Agirre et al., 2015; Barad et al., 2015; Lutteke et al., 2005; Williams et al., 2018). Binding surface area calculations were performed using jsPISA (Krissinel, 2015).

Enzymatic Release of N-linked Glycans

N-linked glycans were released from BG505 SOSIP proteins by in-gel digestion with PNGase F (New England Biolabs). Proteins were resolved by SDS–PAGE and stained with Coomassie Blue. Following destaining, the protein band was excised and washed alternatively with acetonitrile and water. Gel bands were then incubated with PNGase F for 16 h at 37°C. Released glycans were eluted from the gel with water and dried in a SpeedVac concentrator.

Fluorescent Labeling of N-linked Glycans

Released glycans were fluorescently labeled with procainamide (Abcam). Dried glycans were resuspended in 30 μL water before addition of 80 μL labeling mixture: 110 mg/mL procainamide, 60 mg/mL sodium cyanoborohydride in a solution of 70% dimethyl sulfoxide, 30% acetic acid. Samples were incubated at 65°C for 4 h. Labeled glycans were purified using Spe-ed Amide-2 cartridges (Applied Separations).

HILIC-UPLC Analysis of the N-linked Glycans

Fluorescently labeled glycans were separated by HILIC-UPLC on a Waters ACQUITY H-Class instrument using a 2.1 mm × 100 mm Glycan BEH Amide Column (1.7 μm particle size; Waters). The following gradient was run: time=0 min (t=0): 22% A, 78% B (flow rate of 0.5 mL/min); t=38.5: 44.1% A, 55.9% B; t=39.5: 100% A, 0% B (0.25 mL/min); t=44.5: 100% A, 0% B; t=46.5: 22% A, 78% B (0.5 mL/min), where solvent A was 50 mM ammonium formate, pH 4.4, and solvent B was acetonitrile. Fluorescence was measured using an excitation wavelength of 310 nm and a detection wavelength of 370 nm.

Oligomannose-type Glycan Quantification

Quantification of oligomannose-type glycans was measured by digestion with Endo H, which cleaves oligomannose- (and hybrid-) type glycans, but not complex-type (New England Biolabs). Labeled glycans were resuspended in water and digested with Endo H for 16 h at 37°C. Digested glycans were cleaned using a PVDF protein-binding membrane plate (Merck Millipore) prior to HILIC-UPLC analysis as above. The abundance of oligomannose-type glycans was calculated, as a relative percentage, by integration of the HILIC-UPLC chromatograms before and after Endo H digestion, following normalization.

Ion Mobility-Mass Spectrometry of N-linked Glycans

To guide subsequent glycopeptide analyses, we performed IM-MS on a separate, unlabeled aliquot of PNGase F-released glycans from the BG505 SOSIP.664 protein. Glycan compositions were determined using traveling wave IM-MS measurements performed on a Synapt G2Si instrument (Waters, Manchester, UK). The glycan sample was cleaned with a Nafion 117 membrane and a trace amount of ammonium phosphate was added to promote phosphate adduct formation. Glycans were analyzed by nano-electrospray with direct infusion with the following settings: capillary voltage, 0.8–1.0 kV; sample cone, 100 V; extraction cone, 25 V; cone gas, 40 L/h; source temperature, 150°C; trap collision voltage, 4–160 V; transfer collision voltage, 4 V; trap direct current bias, 35–65 V; IMS wave velocity, 450 m/s; IMS wave height, 40 V; trap gas flow, 2 mL/min; IMS gas flow, 80 mL/min. Data were acquired and processed

with MassLynx v4.1 and Driftscope version 2.8 software (Waters, Manchester, UK). Structural assignments were based on previously described IM-MS of BG505 SOSIP.664 glycans (Behrens et al., 2016).

Reduction, Alkylation and Digestion of Env Proteins

BG505 SOSIP proteins (100-150 μg each) were buffer exchanged using Vivaspin 100 kDa columns, denatured, reduced, and alkylated by sequential 1 h incubations at room temperature (RT) in the following solutions: 50 mM Tris/HCl, pH 8.0 buffer containing 6 M urea and 5 mM dithiothreitol (DTT), followed by the addition of 20 mM iodacetamide (IAA) for a further 1h at RT in the dark, and then additional DTT (20 mM), to eliminate residual IAA. The proteins were then buffer-exchanged into 50 mM Tris/HCl, pH 8.0 using Vivaspin 3 kDa columns and aliquots were digested with trypsin or chymotrypsin (Mass Spectrometry Grade, Promega) at a ratio of 1:30 (w/w) for 16 h at 37°C. The reactions were dried and glycopeptides were extracted using C18 Zip-tip (Merck Millipore) following the manufacturer's protocol. Briefly, tips were equilibrated by alternating in acetonitrile and 0.1% trifluoracetic acid. The reaction mixture was loaded on to the tip and eluted with 50% acetonitrile, 0.1% trifluoracetic acid.

Liquid Chromatography-Mass Spectrometry Analysis of Glycopeptides

Eluted glycopeptides were dried again and re-suspended in 0.1% formic acid prior to mass spectrometry analysis. An aliquot of glycopeptides was analyzed by LC-MS with an Easy-nLC 1200 system coupled to an Orbitrap Fusion mass spectrometer (Thermo Fisher Scientific) using higher energy collisional dissociation (HCD) fragmentation. Peptides were separated using an EasySpray PepMap RSLC C18 column (75 μm x 75 cm) with a 275 minute linear gradient consisting of 0%–32% acetonitrile in 0.1% formic acid over 240 minutes followed by 35 minutes of 80% acetonitrile in 0.1% formic acid. The flow rate was set to 200 nL/min. The spray voltage was set to 2.8 kV and the temperature of the heated capillary was set to 275°C. HCD collision energy was set to 50%, appropriate for fragmentation of glycopeptide ions. Glycopeptide fragmentation data were extracted from the raw file using Byonic™ (Version 2.7) and Byologic™ software (Version 2.3; Protein Metrics Inc.). The glycopeptide fragmentation data were evaluated manually for each glycopeptide; the peptide was scored as true-positive when the correct b and y fragment ions were observed along with oxonium ions corresponding to the glycan identified. The relative abundance of each glycan at each site was calculated using the extracted ion chromatograms for true-positive peptides; site-specific glycan compositions for all proteins analysed in this study are given in Table S4.

Site-Specific Glycan Classification

Remaining glycopeptides were first digested with Endo H to cleave oligomannose- and hybrid-type glycans, leaving a single GlcNAc residue at the corresponding site. The reaction mixture was then dried and resuspended in a mixture containing 50 mM ammonium bicarbonate and PNGase F using only ^{18}O-labeled water (Sigma-Aldrich) throughout. This second reaction cleaves the remaining complex-type glycans, leaving the GlcNAc residues intact. The use of $H_2{}^{18}O$ in this reaction enables complex glycan sites to be differentiated from unoccupied glycan sites as the hydrolysis of the glycosidic bond by PNGase F leaves an ^{18}O isotope on the resulting aspartic acid residue. The resultant peptides were purified by C18 ZipTip, as outlined above, and subjected to LC-MS in a similar manner to before, but using a lower HCD energy of 27% as glycan fragmentation was not required. Data analysis was performed as above.

ELISAs

High binding 96 well assay plates (Corning) were incubated with BG505 SOSIP.664 proteins (10 μg/mL in PBS) overnight at 4°C. Plates were washed with a solution of PBS containing 0.5% Tween 20 (v/v) and blocked for 1h at RT with 5% milk in PBS + 0.5% Tween. After another wash step, the primary antibody was incubated (1:2 dilution series with a starting concentration of 20 μg/mL) in PBS for 1h at RT. Plates were washed and an anti-human IgG conjugated to Horseradish Peroxidase (Abcam) secondary antibody was added at a 1:2000 dilution in PBS. Plates were washed and TMB substrate solution (Thermo Fisher Scientific) was added. The reaction was stopped with sulfuric acid after 5 min and the OD 450 nm was measured.

Biolayer Interferometry

BG505 SOSIPv4.1 mutants with C-terminal 2XStrep tags were expressed and purified by PGT145 affinity chromatography as described above. 25 μg/mL SOSIP with C-terminal 2XStrep tag in kinetics buffer (PBS, pH 7.4, 0.01% [w/v] BSA, and 0.002% [v/v] Tween 20) were loaded onto Streptavidin biosensors (Fortébio) and dipped into wells containing a seven-step, 2-fold dilution series of 2G12 Fab_2 starting at 2000 nM. Kinetic parameters were calculated with the Octet Data Analysis software (Fortébio) using the 1:1 model association/disassociation model.

QUANTIFICATION AND STATISTICAL ANALYSIS

The integration of peaks corresponding to fluorescently labeled N-glycans was performed using Empower 3.0 (Waters, Manchester, UK) (Figures 2 and S3). The IM-MS data used to generate the glycan library were acquired and processed with MassLynx v4.1 and Driftscope version 2.8 software (Waters, Manchester, UK) (Figure S4). Chromatographic areas were extracted for site-specific analysis using Byonic™ (Version 2.7) and Byologic™ software (Version 2.3) by Protein Metrics (Figures 3–5, S2, and S5).

Structure, Volume *28*

Supplemental Information

Networks of HIV-1 Envelope Glycans Maintain Antibody Epitopes in the Face of Glycan Additions and Deletions

Gemma E. Seabright, Christopher A. Cottrell, Marit J. van Gils, Alessio D'addabbo, David J. Harvey, Anna-Janina Behrens, Joel D. Allen, Yasunori Watanabe, Nicole Scaringi, Thomas M. Polveroni, Allison Maker, Snezana Vasiljevic, Natalia de Val, Rogier W. Sanders, Andrew B. Ward, and Max Crispin

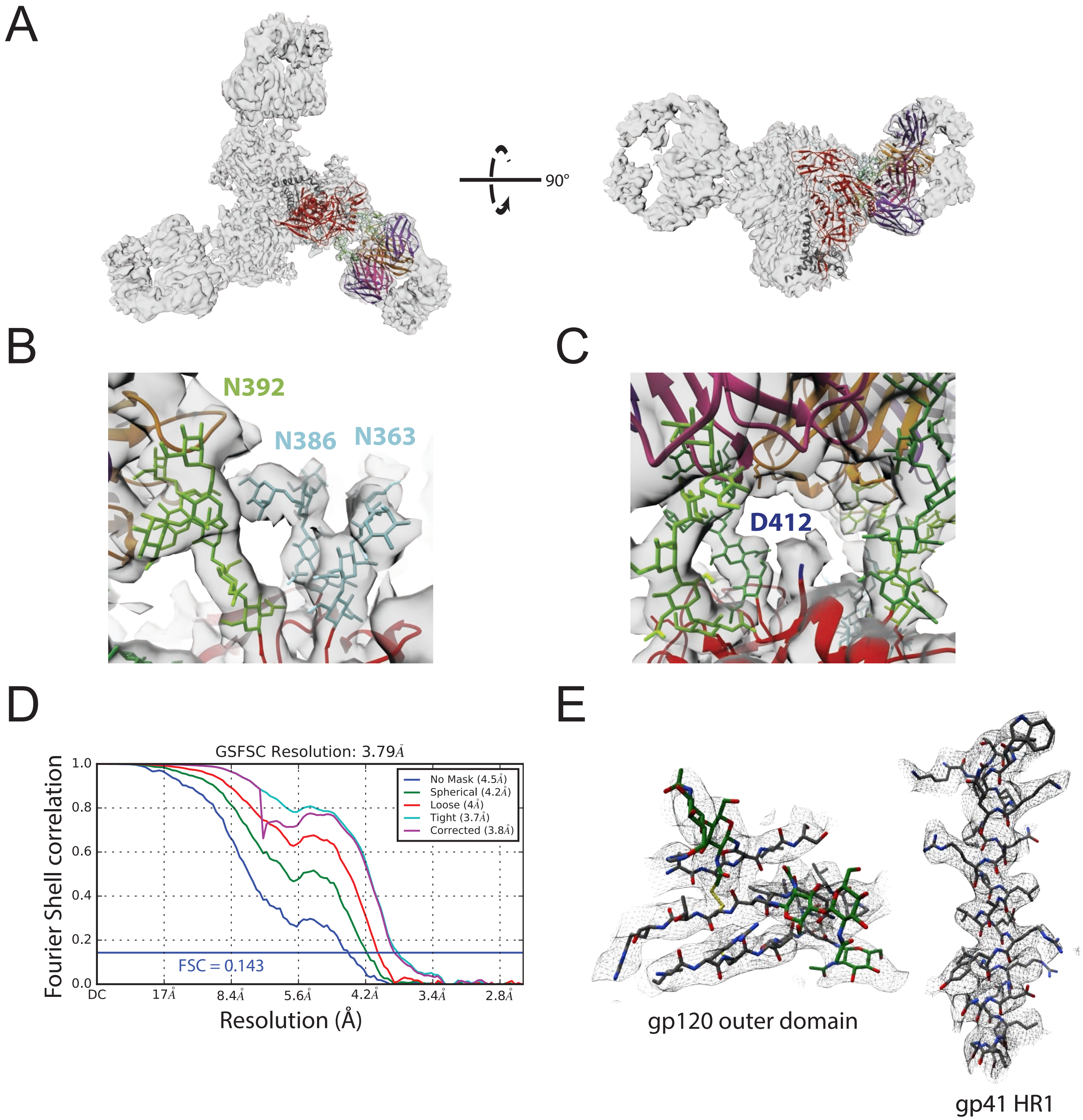

Figure S1. Cryo-EM of BG505 SOSIP.664 and 2G12 Fab2. Related to Figure 1. A) 3.8 Å cryo-EM 3D reconstruction and refined atomic model of BG505 SOSIP.664 with 2G12 Fab2. B) The close proximity of the N363 and N386 glycans to the N392 glycan could provide support for 2G12 binding via glycan/ glycan interactions. C) No coordinated density was seen for the N411 amino acid or the associated N- linked glycan. D) Fourier shell correlation (FSC) curves calculated in cryoSPARC during final non- uniform refinement. E) EM densities with atoms shown for a beta sheet in the gp120 outer domain and an alpha helix for the gp41 HR1 region. Related to Figure 1.

A

	N88	N130	N133	N136	N137	N141	N156	N160	N185e	N187	N188	N190	N190c	N197	N230	N234	N241	N262	N276	N289	N295	N301	N332	N339	N355
A	99	47	14	11	22	17	98	94	7	33	6	4	12	99	3	80	96	100	97	22	54	94	66	63	5
B	99	73	10	23	25	17	96	89	6	22	45	2	8	98	26	72	97	100	97	67	79	95	85	70	4
C	98	54	28	9	25	12	95	92	6	41	8	2	8	98	78	84	97	98	92	79	20	95	77	71	4
D	98	71	3	22	32	9	91	94	10	34	38	3	17	98	17	84	95	99	91	87	76	73	80	75	1
F	98	52	13	10	25	16	95	90	6	29	8	2	11	98	10	83	95	100	98	94	95	95	75	79	6
G	98	62	27	11	28	11	99	94	4	27	23	2	21	99	4	80	97	99	97	79	48	98	70	16	5
AE	98	66	14	3	14	16	98	93	8	39	6	2	6	98	0	86	99	100	95	96	85	79	2	47	4
Total	99	64	16	15	24	15	96	91	6	31	26	2	9	98	37	78	97	99	95	72	60	93	72	67	4

	N356	N362	N363	N386	N392	N396	N398	N406	N407	N411	N442	N448	N461	N462	N611	N616	N618	N625	N637
A	82	22	42	85	82	22	39	5	5	51	6	93	27	30	98	95	2	95	96
B	82	59	5	83	83	32	38	7	6	42	4	92	22	30	99	89	7	97	97
C	74	19	3	88	72	24	27	4	4	44	73	74	18	29	98	92	4	97	97
D	17	10	3	91	79	22	20	7	6	38	2	98	27	39	99	95	2	94	93
F	84	62	0	84	73	2	8	0	0	65	43	71	16	37	100	95	0	100	95
G	32	52	0	92	68	8	22	0	0	49	59	87	28	32	100	95	2	97	98
AE	75	1	0	93	77	15	43	1	1	64	7	95	31	25	98	98	0	97	98
Total	76	37	6	86	78	26	34	5	5	46	27	87	22	29	99	92	5	97	97

B

	Mean
A	28.9
B	30.5
C	29.1
D	29.2
F	29.7
G	30.0
AE	28.6

0 – 100 %

C

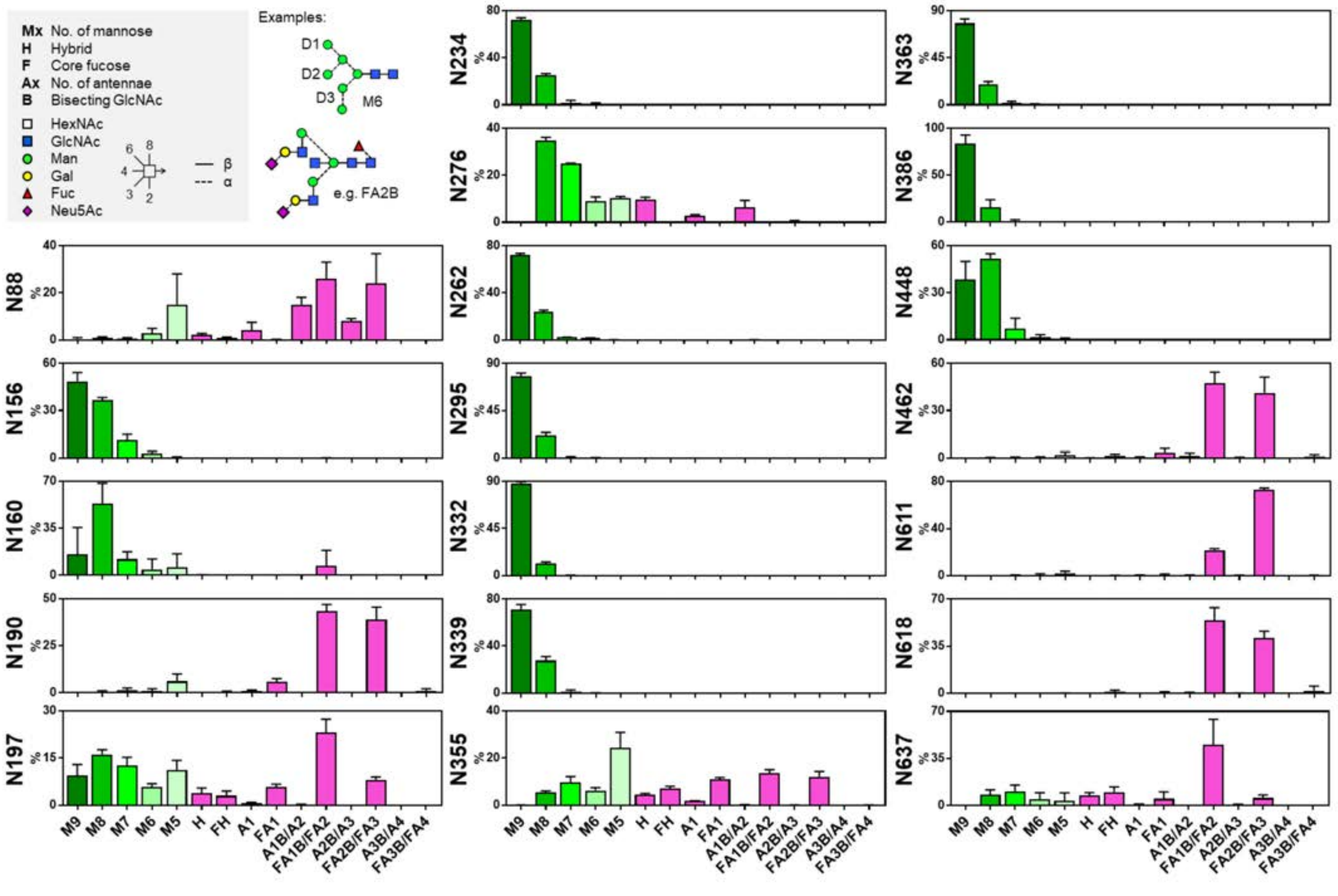

Figure S2. Conservation of PNGS across clades and reproducibility of LC-MS glycopeptide analysis. Related to Figures 1, 3, 4 and 5. A) Percentage conservation of individual PNGS. B) Mean number of total PNGS. The analysis was performed using sequence data obtained from the 2017 filtered web alignment from the Los Alamos HIV sequence database (https://www.hiv.lanl.gov). Clade A, n = 338; clade B, n = 2351; clade C, n = 1524; clade D, n = 127; clade F, n = 63; clade G, n = 114, clade AE, n = 535. Only the second position Asn was included for NN[S/T][S/T] motifs. Sites are only shown if present in one of the following representative strains: BG505 (clade A), AMC011 (clade B), CZA97 (clade C). The low conservation of the N332 site in clade AE is compensated by the high use of the N334 site (92%, AE; 21%, total). C) Relative quantification of glycan sites from three biological replicates of BG505 SOSIP.664. Error bars represent the standard error of the mean. The inset key depicts the glycan nomenclature and classification system used throughout this manuscript. HexNAc = N-acetylhexosamine, GlcNAc = N-acetylglucosamine, Man = mannose, Gal = galactose, Fuc = fucose, Neu5Ac = sialic acid. Glycan nomenclature according to the Consortium for Functional Glycomics, with linkages as per the Oxford system. Related to Figure 2.

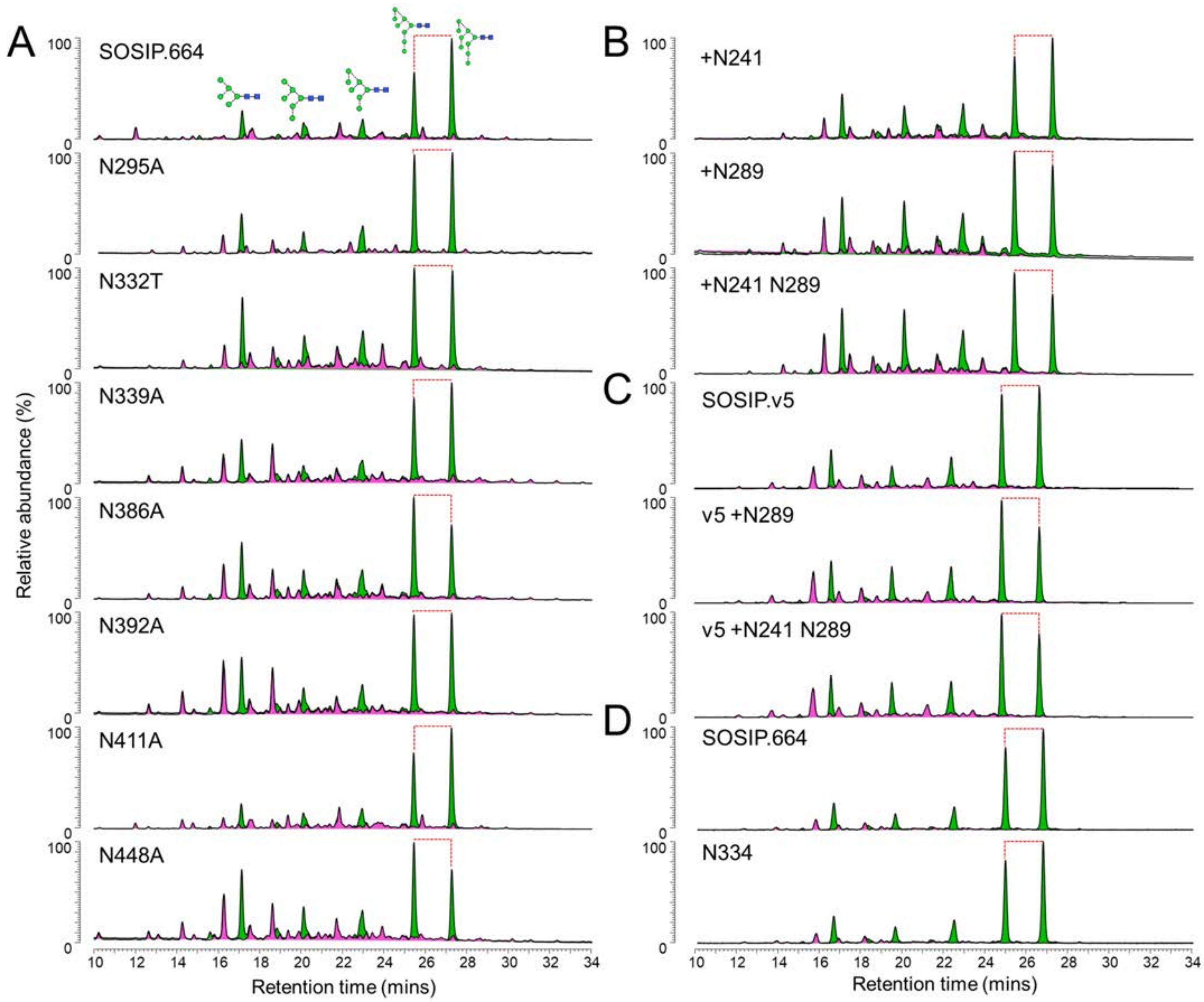

Figure S3. Glycosylation profiles of glycan mutants. Related to Figure 2. Hydrophilic interaction liquid chromatography-ultra performance liquid chromatography of PNGase F-released N-linked glycans from A) BG505 SOSIP.664 knockouts, B) BG505 SOSIP.664 knock-ins, C) BG505 SOSIP.v5 glycan knock-ins, and D) N334 shift mutation. Oligomannose-type glycans (judged by sensitivity to Endo H digestion) are highlighted in green. Complex-type glycans are shown in pink. Related to Figure 2.

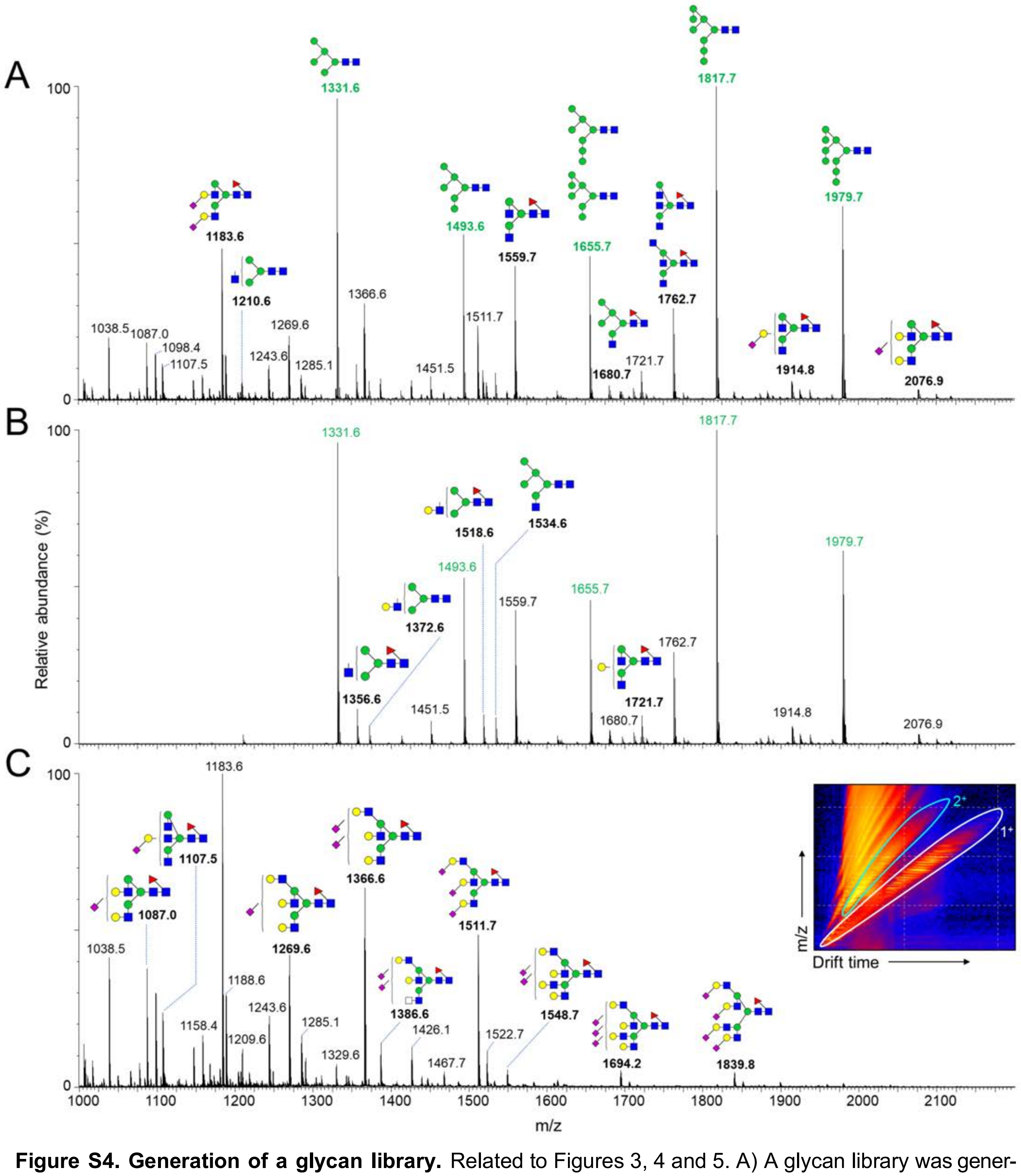

Figure S4. Generation of a glycan library. Related to Figures 3, 4 and 5. A) A glycan library was generated by negative ion electrospray ion mobility-mass spectrometry of an aliquot of unlabelled N-glycans from BG505 SOSIP.664 trimers. B) Mobility-extracted singly charged negative ions. C) Mobility-extracted doubly charged negative ions. The corresponding ions are encircled in white and blue, respectively, in the inset ion mobility drift plot. Related to Figure 3.

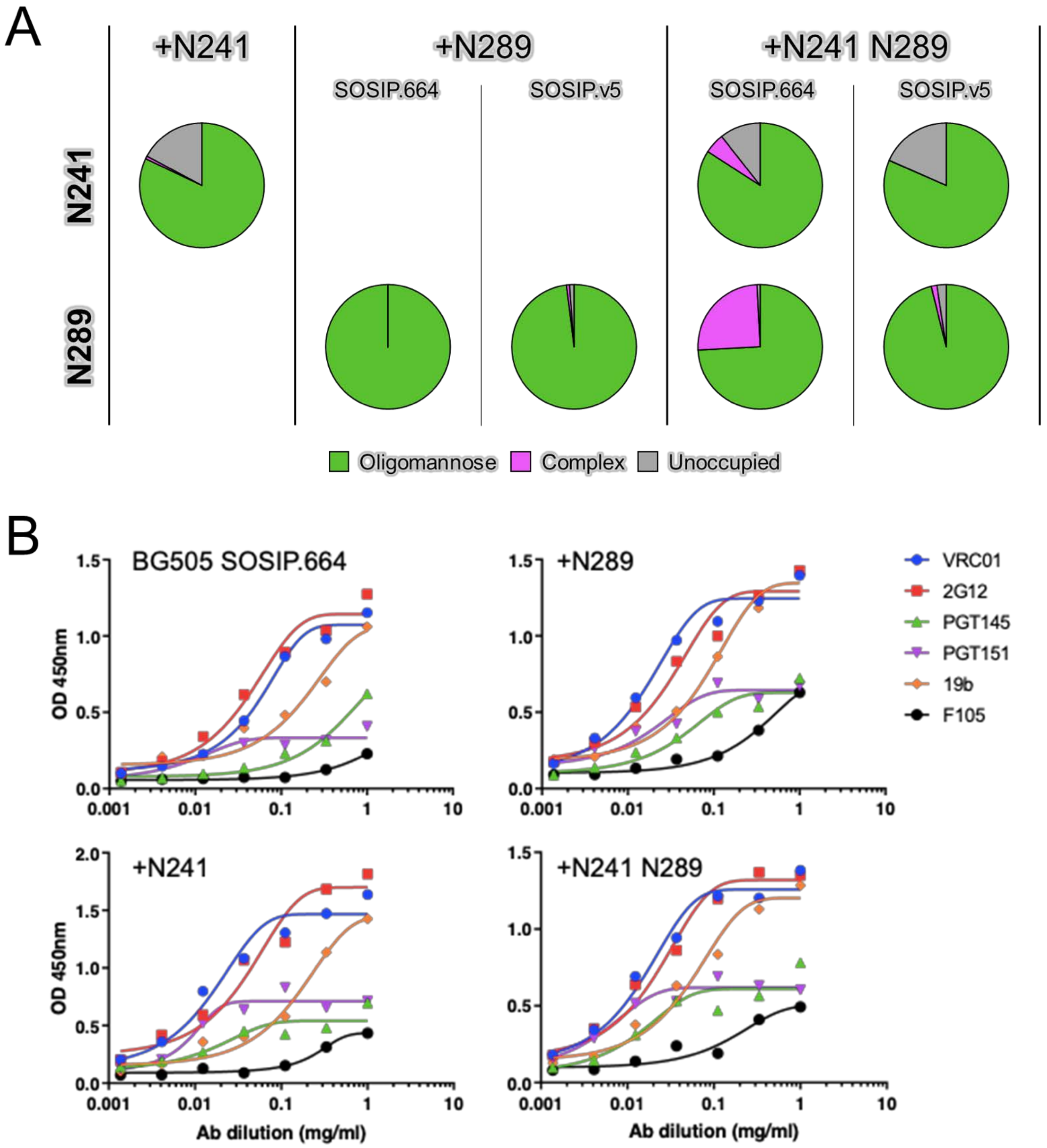

Figure S5. Classification of glycan-type at the N241 and N289 sites. Related to Figure 4. A) Relative quantification of the glycan-type occupying the N241 and N289 sites in the SOSIP.664 and SOSIP.v5 knock-in mutants. Glycopeptides were digested with Endo H to cleave oligomannose-type glycans, leaving behind a GlcNAc residue. The remaining complex-type glycans were then digested with PNGase F in the presence of ^{18}O-labeled water, to convert the Asn to an ^{18}O-labeled Asp, prior to LC-MS analysis, B) The BG505 SOSIP.664 glycan knock-ins are antigenically similar, as assessed by ELISA binding to a panel of neutralizing antibodies. Related to Figure 4.
